# Context-dependent dynamics lead to the assembly of functionally distinct microbial communities

**DOI:** 10.1038/s41467-020-15169-0

**Published:** 2020-03-18

**Authors:** Leonora S. Bittleston, Matti Gralka, Gabriel E. Leventhal, Itzhak Mizrahi, Otto X. Cordero

**Affiliations:** 1 Department of Civil and Environmental Engineering, Massachusetts Institute of Technology (MIT), Cambridge, MA USA; 2 Department of Biological Sciences, Boise State University, Boise, ID USA; 3 Department of Life Sciences and the National Institute for Biotechnology in the Negev, Ben-Gurion University of the Negev, Beersheba, Israel

**Keywords:** Community ecology, Microbial communities

## Abstract

Niche construction through interspecific interactions can condition future community states on past ones. However, the extent to which such history dependency can steer communities towards functionally different states remains a subject of active debate. Using bacterial communities collected from wild pitchers of the carnivorous pitcher plant, *Sarracenia purpurea*, we test the effects of history on composition and function across communities assembled in synthetic pitcher plant microcosms. We find that the diversity of assembled communities is determined by the diversity of the system at early, pre-assembly stages. Species composition is also contingent on early community states, not only because of differences in the species pool, but also because the same species have different dynamics in different community contexts. Importantly, compositional differences are proportional to differences in function, as profiles of resource use are strongly correlated with composition, despite convergence in respiration rates. Early differences in community structure can thus propagate to mature communities, conditioning their functional repertoire.

## Abstract (web summary)

Historical contingency can affect community composition and function, but the extent to which this occurs is unclear. Here the authors use pitcher plant microbial communities to demonstrate that community dynamics and key metabolic functions at equilibrium depend on history and initial composition.

## Introduction

Microbes profoundly shape our ecosystems, yet we still lack a clear understanding of the processes driving community assembly and related ecosystem functioning^1^. Community assembly is difficult to predict, because the dynamics of any particular species can be dependent on the community context; niches are created or destroyed through biotic interactions with other members of an assembling community^2–4^. Microbial communities are complex, with many species and different kinds of interactions among species. For example, microbes can facilitate other species’ growth via excretion of metabolic waste products^5–8^, or actively interfere with their growth through the production of antimicrobial compounds^9^. Microbes can also engage in strong cooperative interactions, whereby energy transducing metabolic interactions are coupled across species^10^. This diversity of interactions creates many potential contexts for species dynamics, implying that the behavior of one species is dependent on the background of interactions. Stochastic changes in the biotic context—for example, priority effects and random colonization or extinction events—can have long-lasting consequences for community structure^3^. As a result, microbial communities can reach different compositional states due to variation in biotic context in addition to variation in abiotic environmental conditions such as weather events or resource pulses. These history-dependent effects are collectively called “historical contingencies”^2–4^.

The extent to which historical contingency leads to alternative community states^2,11^ remains an active subject of debate. In a strongly selective environment, historical contingencies may not matter and communities converge to the same compositional and functional outcomes^12^. For example, bacterial communities from widely different sources converged reproducibly in single-carbon source synthetic media^7^. Beyond microbes, Mediterranean plant communities in environments with frequent fires were formed by related groups of species that share key traits^13^. In contrast, other studies have found that historical contingency leads to different community compositions and functions, for example: priority effects led to large differences in ecosystem function for wood-decaying fungi^14^ and productivity of grassland plants^15^. A third, and perhaps largest, set of studies has found convergence in terms of function but not species (or phylogenetic) composition, for example: grassland plants^16^, the stratified layers of a hypersaline microbial mat^17^, microbial communities colonizing the surface of seaweed^18^, and the bacteria and archaea living in bromeliad tanks^19^.

Functional convergence without species convergence is more likely when the functions being measured are performed by many species from different lineages, and thus are redundant within the broader species pool. Here, again, there are contrasting reports in the literature on the prevalence of functional redundancy. A number of studies have found functional redundancy in microbial communities^19,20^, while others emphasize important functional differences that depend on species composition^21^. The degree of redundancy is clearly related to the function and system examined; for example, aerobic respiration is found across many bacteria, while the ability to degrade lignin is rare. Thus, “narrow” functions, such as the hydrolysis of complex carbon compounds, are generally carried out by rare community members^22^. When relevant functions are variable across genetic backgrounds (not highly redundant) and dependent on interactions, historical contingencies could have major effects on the functional capabilities of a community and on nutrient cycling within ecosystems^14^. Therefore, studies in microbial ecology need to address more specific, relevant functional measurements and examine not just historically contingent compositional states, but also historically contingent functional states.

The modified leaves of carnivorous pitcher plants host small ecosystems composed of bacteria, fungi, protozoa, rotifers and arthropods^23,24^ and present an excellent system to investigate the effects of historical contingency on function and composition. Bacteria, in particular, are thought to assist their pitcher plant hosts in breaking down captured prey^25–28^, creating a clear link between a relevant ecosystem function, the degradation of insect material, and the microbial species composition. The degradation of chitin, a primary component of insect exoskeletons, is particularly relevant in our study, as chitinase enzymes are not produced by the host plant and thus only the chitinases excreted by the associated microbial community can breakdown this complex biopolymer. Using bacterial communities from ten wild pitchers of the purple pitcher plant, *Sarracenia purpurea*, we test to what extent historical contingencies impact community assembly and substrate degradation. To this end, we transfer the communities from living pitcher plants into in vitro microcosms and perform a serial transfer experiment, whereby communities are serially passaged until a stable composition is reached. During this assembly process, microcosms can converge or remain distinct due to differences in the initial species pool as well as to differences in biotic interactions across the microcosms. By comparing the assembly dynamics of 10 different plant microbiomes we ask to what extent communities converge to a single compositional and functional state, and whether any lack of convergence could be explained by historical contingency.

## Results

### Distinct, stable communities assemble in microcosms

**Fig. 1:**
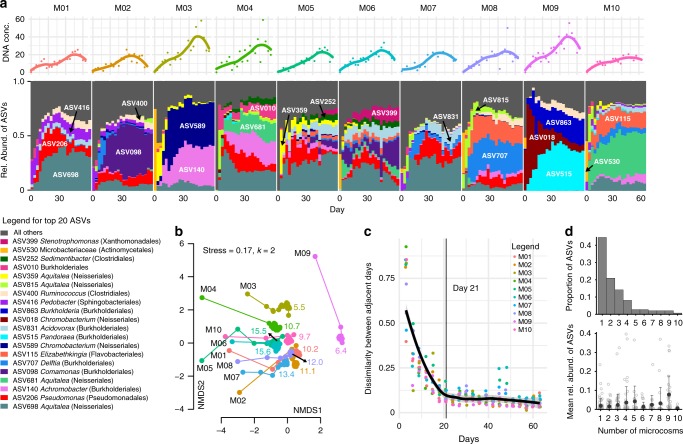
Microcosm communities approach distinct equilibria. **a** Relative abundances of the top 20 Amplicon Sequence Variants (ASVs) across the ten microcosms during the course of the serial transfer experiment. ASVs are listed one time each on the bar plot, with taxonomic classification in the legend. The DNA concentrations for each timepoint are graphed above as points, with a Loess fit as a solid line. **b** Communities change quickly and then stabilize in the two-dimensional Non-metric Multidimensional Scaling (NMDS) plot of Bray–Curtis dissimilarities of the microcosm community compositions. The microcosm name is listed in black next to the Day 0 point for each microcosm, and the lines connect the timepoints. Colored numbers indicate the mean effective number of species for each community post Day 21. **c** The Bray–Curtis dissimilarity of ASV relative abundances between adjacent days decreases over the course of the experiment. The thick black line shows a Loess fit to all data points, and the thin line marks Day 21. **d** At the end of the experiment, most ASVs were present in only one microcosm (top), but ASVs present in more microcosms tended to have higher mean relative abundances (bottom), shown as black circles with ±1 standard deviation error bars.

The aquatic communities from 10 individual *Sarracenia purpurea* pitchers were filtered to focus on bacteria and inoculated into a realistic, complex nutrient source: sterilized, ground crickets in acidified water. The in vitro communities were serially transferred every 3 days for 21 transfers, using a low dilution rate of one-part culture to one-part fresh media. We repeated the process with the unfiltered communities as well. Community composition was measured for each transfer using 16S rRNA sequencing (see Methods). From ~8 million sequences across all microcosms and timepoints, DADA2 analysis inferred 889 distinct ASVs (Amplicon Sequence Variants, which we treat as units of diversity). The most abundant phylum was Proteobacteria, followed by Firmicutes and Bacteroidetes. The top twenty ASVs are displayed in Fig. 1a, accounting for 69.4% of the reads; for ASVs without assigned genera, genus names were recovered from the full 16S rRNA genes of our cultured strains that matched 100% with ASV sequences. The most abundant genera (*Aquitalea*, *Pseudomonas*, *Achromobacter*, *Comamonas*, and *Delftia*), all contain bacterial species known to live in freshwater, soil, or plant-associated habitats^29–33^. Using DNA concentrations as a proxy to measure biomass increase, we found that there is a ~10-fold increase in biomass during in vitro assembly (Fig. 1a). Microcosms M03 and M09 stood out as those with the highest biomass yield as well as having the lowest diversity.

Community assembly dynamics in our microcosms were dominated by both ASV loss and dramatic changes in ASV abundances. We assessed coarse-grained dynamics using the Bray–Curtis dissimilarity between subsequent time points, which exhibited two seemingly distinct phases (Fig. 1b, c): a first phase of rapid change, where many species went extinct and the survivors increased in frequency; and a second phase of slow changes that started after seven transfers (21 days, indicated by the thin line in Fig. 1c), in which extinctions were rare and likely caused by competitive interactions. In this last phase, communities slowly approached what seemed to be an equilibrium state (Fig. 1b, c). However, the near-equilibrated communities maintained significant differences both in terms of composition and diversity. For example, no microcosm had the same top three ASVs (Fig. 1a), the NMDS ordination showed generally non-overlapping points for the different microcosms (Fig. 1b), and, even after Day 21 the effective number of species (see Methods for calculation) still differed among the microcosm communities, ranging from ~6 to 16 (small colored numbers in Fig. 1b). Most ASVs were rare (~65% found in ≤2 microcosms) and had low mean relative abundance (<1%), but the few core ASVs found across nine microcosms had higher mean relative abundances (~10%, Fig. 1d). Community composition at the family level remained distinct across our 10 microcosms, and phylogenetic metrics of alpha and beta diversity showed the same dynamics as non-phylogenetic metrics, indicating that observed differences were not simply due to changes in closely related species (Supplementary Fig. 1). In summary, communities first approached convergence due to the fast loss of diversity (primarily along the first NMDS axis), but remain distinct due to historical contingencies (primarily along the second NMDS axis).

**Fig. 2:**
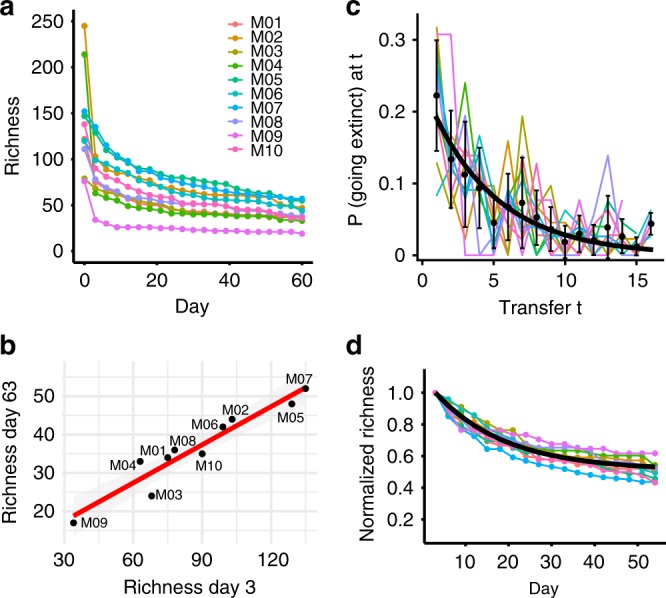
Early richness predicts final richness and communities equilibrate at a common rate. **a** Richness over time for each microcosm community. **b** Richness on Day 3 (1st timepoint) is strongly correlated with richness on Day 63 (21st timepoint). Linear model: *R*^2^ = 0.9008, *p* = 1.714 × 10^−5^. **c** The probability of going extinct at transfer *t*. Colored lines are probability densities for individual microcosms. Black points are averages across microcosms, the black line is the maximum likelihood distribution with a common parameter across microcosms (see main text and Methods) given by the inverse mean extinction time. **d** Richness over time for each microcosm community, normalized by the richness on Day 3. The black line shows the exponential decay curve parametrized by the mean proportion of surviving ASVs (from **b**) and the common ASV extinction rate (from **c**).

Interestingly, the differences in richness near-equilibrium were seemingly pre-determined at early stages of assembly. Communities lost many ASVs between days 0–3 during initial adjustment to the laboratory environment (Fig. 2a), but the richness measured from the first timepoint of the experiment (Day 3) was an excellent predictor of the richness at the end of the experiment (Day 63) in a linear model with *R*^2^ = 0.9008 and *p* < 0.0001 (Fig. 2b). Notably, richness in samples taken directly from the pitcher plant (Day 0) had no significant correlation with that of Day 63 (linear model, *R*^2^ = 0.1978, *p* = 0.1105), consistent with the notion that some ASVs in the pitcher plant fluid were either metabolically inactive or unable to grow in our experimental conditions. Thus, changes in community composition after only three days of adjusting to the lab environment propagated throughout the assembly process, suggesting that historical contingencies played a significant role in structuring communities.

We found that the effects of historical contingency on community assembly were remarkably consistent and reproducible, implying that the lack of convergence was not due to stochastic dynamics but to deterministic effects. We performed the same experiment on a second set of microcosms from the same inocula that were not subjected to initial filtering, but otherwise underwent the same transfer protocol and amplicon sequencing (Supplementary Fig. 2). These microcosms were very similar to their filtered counterparts in composition, DNA concentrations, community dynamics over time and the equilibration process (Supplementary Fig. 2a–c). Indeed, unfiltered and filtered microcosms were indistinguishable in an NMDS visualization of their assembly dynamics (Supplementary Fig. 2b). Furthermore, in the unfiltered samples we saw the same strong correlation between the richness on Day 3 and 63 (Supplementary Fig. 2d). Taken together, these results reinforce the notion that initial pitcher source environment and composition predetermine the future states of community assembly.

Given the strong correlation between initial and final richness, we wondered whether temporal dynamics of community equilibration were also correlated across microcosms. For example, the temporal dynamics of ASV loss could be driven by external factors such as transfer intervals and the dilution factor, in which case all microcosms should exhibit comparable dynamics. Alternatively, the dynamics might be driven by community context and thus specific to each microcosm. To answer this question, we measured the distribution of ASV extinction times across the microcosm communities (Fig. 2c). As a null model, we tested if the loss of ASVs is a random process where each ASV that is bound to go extinct in a given community context does so with a fixed probability *p* in each transfer. This assumption implies that the extinction time distribution is described by a geometric distribution, which indeed gave a good fit for all microcosms (Pearson’s chi-squared test for differences was not significant: *p* > 0.05 in all cases). To investigate if microcosms can be described by a common extinction time distribution, we used the Bayesian Information Criterion to compare two models: using either one parameter per microcosm or a single parameter describing all microcosms. Surprisingly, the single-parameter model was strongly favored (relative likelihood = 7.7 × 10^10^) indicating that, despite biotic differences in richness and composition across microcosms, there was a common rate of ASV extinction. The process of ASV loss in unfiltered communities was also well-described by a geometric distribution using a single parameter to describe all microcosms (relative likelihood = 1.5 × 10^9^, Supplementary Fig. 2e). This “universal” dynamic of species loss was also revealed when studying relative richness, i.e. normalized by richness at Day 3 (Fig. 2d). After this normalization, all relative richness curves mapped well to our model’s maximum likelihood distribution and approached a common equilibrium relative richness level (~50% of the initial richness, regardless of its absolute value, Fig. 2d). Thus, about half of all initial ASVs within a given microcosm were doomed to go extinct at a random point during the assembly process, while the rest persisted indefinitely. Interestingly, this result has been predicted by theory. Models based on random interaction matrices indeed predict that that the richness of the community after assembly is a fixed proportion of the initial richness of the species pool^34^. Our work is the first empirical validation of this prediction.

**Fig. 3:**
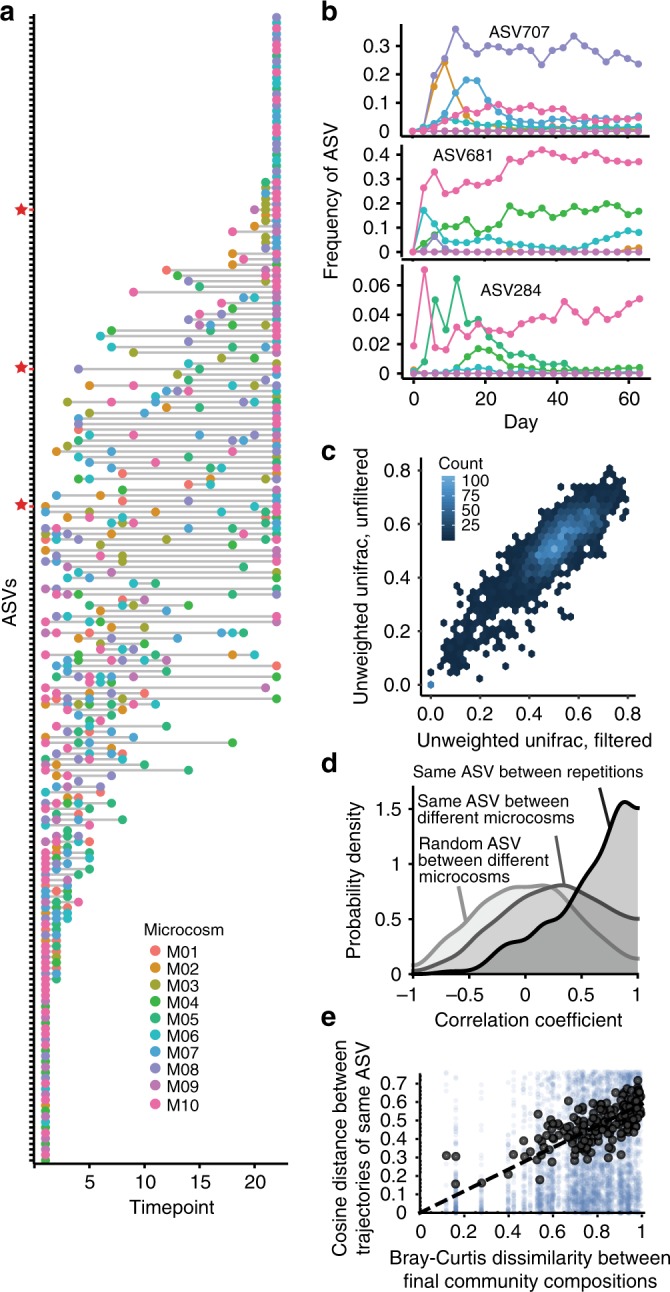
Species dynamics are context dependent. **a** The *y*-axis lists all ASVs shared by at least two microcosms. Colored points mark which day the ASV was lost from that particular microcosm. Points at the far right of the graph are ASVs that persisted to the end of the serial transfer experiment. Red stars mark the ASVs shown in **b**. **b** Three examples of ASV dynamics in the different microcosms over time, where ASVs were lost early in some microcosms but persisted until the end in others. **c** Unweighted UniFrac distances in filtered and unfiltered communities (repetitions) are strongly correlated, highlighting that community dynamics are reproducible given the same inoculum. Mantel test, *r* = 0.85, *p* = 1 × 10^−4^; Linear model, *R*^2^ = 0.78, *p* = 2.2 × 10^−16^. Samples from Days 0–21 are included here, to capture dynamics up until equilibration. Hexplot color indicates the density of values in each hexagon. **d** Probability density of correlation coefficients between the same ASVs in repetitions started from the same inocula, between the same ASVs in distinct microcosms, and between randomly chosen ASVs. Correlation coefficients are large when comparing replicate communities started from the same pitcher plant inocula. Between different microcosms, there is a moderate increase in the number of positive correlation coefficients relative to random pairs. **e** Mean Bray–Curtis dissimilarities between final community compositions is correlated with mean cosine distances between trajectories of the same ASV.

Although microcosms display similar extinction dynamics, the same ASV may still behave differently depending on the community in which it is present. To study the context-dependency of each species, we focus on those shared ASVs, which although representing a small subset of the species list, accounted on average for ~90% of the communities’ relative abundances (Fig. 1d). To ask to what extent species dynamics were dependent on community context, we first looked at the extinction times and found that the majority of the shared ASVs dropped out of different microcosms at different times (Fig. 3a), often with large differences in their persistence time. For example, ASV681 (in Fig. 3b) dropped out by Day 12 in microcosm M08, but persisted at high relative abundance through the end of the experiment in microcosms M04, M06, and M10. Because microcosm compositional dynamics were very similar when starting from the same initial bacterial inoculum (correlation of unweighted UniFrac distances shown in Fig. 3c), we leveraged the unfiltered samples to investigate context dependent behavior of individual ASVs.

We focused on ASVs that persisted at least once past equilibration (Day 21) and found that the temporal trajectories of the same ASVs were strongly dependent on community composition. ASVs present in multiple microcosms that survived the early extinction phase were correlated ~40% of the time across microcosms and were more likely to share the same fate (survive vs. go extinct) than a null model (Supplementary Fig. 3). However, while the cross-microcosm correlations of ASVs were moderately higher than those of any two random ASVs (mean = 0.23 and mode = 0.32 vs. random pairs: mean = 0.003 and mode = 0.01, Fig. 3d), species identity alone was not predictive of their dynamics; the community context was far more important. Indeed, when we compared ASV dynamics between filtered and unfiltered microcosms started from the *same* inoculum, we found much higher correlations (mean = 0.55 and mode = 0.89, Fig. 3d), consistent with the notion that the dynamics in a given community context are deterministic. The same was true when comparing at the family level (Supplementary Fig. 4). More importantly, across all microcosms, similarity in community composition was positively correlated with the cosine similarity (see Methods for details) in ASV dynamics (intercept-free linear model, R^2^ = 0.63, Fig. 3e). This means that biotic context, and not species identity, was the main factor that explained the ASV dynamics across microcosms. Because community composition is the only factor that changes across microcosms, this result implies that biological interactions (e.g. competition, niche construction via secreted metabolites, etc.) are the main drivers of population dynamics.

### ASV dynamics are highly correlated with functional dynamics

**Fig. 4:**
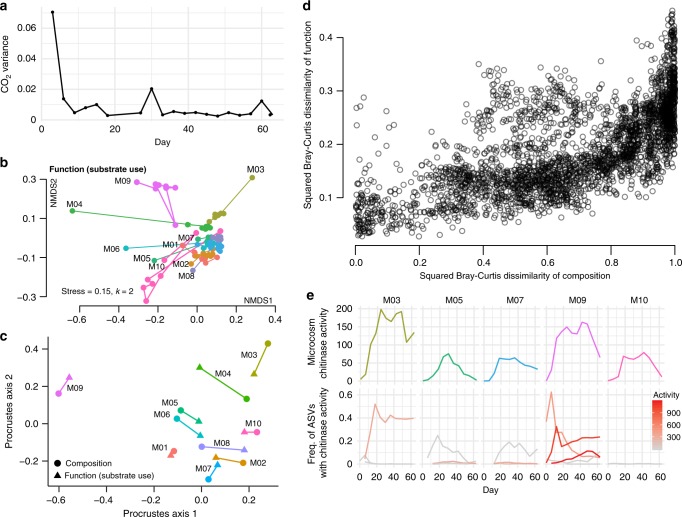
Community composition strongly correlates with functional activity. **a** Variance in percent CO_2_ among the ten microcosms over the course of the serial transfer experiment. **b** Two-dimensional NMDS plot of the Bray–Curtis dissimilarities of functional activity (substrate use) as measured by EcoPlates. The microcosm name is listed in black next to the Day 0 point, and the lines connect the timepoints. **c** Procrustes rotation of the composition NMDS plot with the function NMDS plot for Day 63 (Procrustes correlation = 0.8991, *p* = 0.001). **d** Plot of Bray–Curtis dissimilarities in composition between all measured timepoints across all microcosms compared with the dissimilarities in functional profile for the corresponding samples (Mantel test r = 0.6403, *p* = 1 × 10^−4^), squared to better illustrate the spread of points. **e** Endochitinase activity over time for the five microcosms that strains were cultured from (top row) and frequency of the ASVs over time that map to strains with measurable endochitinase activity (bottom row). Endochitinase activity is shown in units/mL for the strains/ASVs in the bottom row by a gradient from gray (low activity) to red (high activity).

Functional redundancy is thought to be widespread in regional pools of bacteria^35^, and thus communities can have diverse compositions but converge to similar functional activity. Previous studies have generally found stronger convergence in function than in composition, and this was a likely outcome from our experiment as all microcosms experienced the same environment in terms of nutrients, temperature and light. In agreement with this, carbon dioxide production, as measured with the MicroResp system, was highly variable across the different microcosms for the first measurement (Day 0–3), but then quickly converged to a similar level (Fig. 4a). After Day 3, communities could not be reliably distinguished based on their CO_2_ output. The low variation among microcosms in percent CO_2_ suggests that the bacterial communities were respiring at about the same rate.

In contrast with CO_2_ production and the expectation of functional convergence, the stabilized microcosm communities showed clear differences in substrate use, as measured across 31 substrates with Biolog EcoPlates (Supplementary Fig. 5, Fig. 4b, c). For example, M09 was the only community able to metabolize 2-Hydroxybenzoic acid (salicylic acid) but unable to metabolize itaconic acid. These particular substrates may not be relevant in the pitcher plant context, but differences in EcoPlate functional fingerprints suggest a likelihood of other functional differences between microcosms. When plotting an NMDS ordination of Bray–Curtis dissimilarities based on EcoPlate functional measures (Fig. 4b) we found a very similar pattern as with species composition: functional activity undergoes an initial large shift and becomes more similar, yet remains distinct across microcosms. A Procrustes test comparing the NMDS plots of composition and function at the end of the experiment recovers a strong and highly significant correlation: 0.8991, *p* = 0.001 (Fig. 4c). Furthermore, when comparing composition to function across all days and samples using Bray–Curtis dissimilarities, samples with similar composition generally also had similar functional activity (Fig. 4d), and are strongly correlated in a Mantel test (*r* = 0.6403, *p* = 1 × 10^−4^). The correlation is in fact higher when only comparing the final day’s measurements (*r* = 0.6907, *p* = 0.002).

**Fig. 5:**
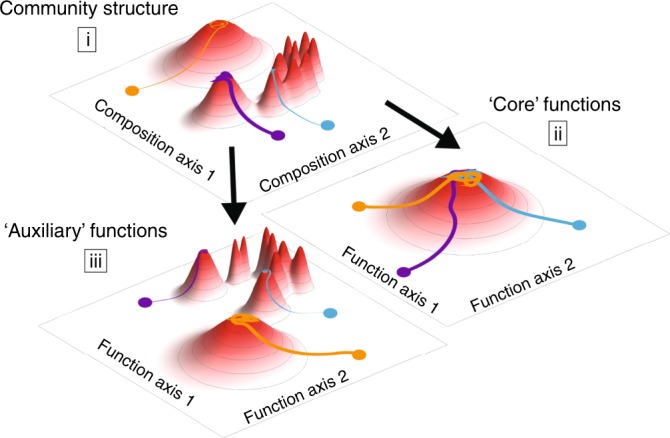
Functional convergence depends on the type of function. Microcosms with the same environmental conditions assemble communities with distinct equilibria due to historical contingencies (i). Core functions such as respiration converge in a common environment, in agreement with the common notion that function and taxonomy are decoupled (ii). However, our results show key functional differences in ‘auxiliary’ metabolic functions, such as chitinase activity or carbon source preference, are strongly coupled to community composition (iii).

To profile the hydrolytic activity of the community, we focused on the activity of chitinases—the enzymes that degrade chitin—since chitin is the main component of insect exoskeletons and is a key carbon and nitrogen source in the pitcher plant system. In two different cricket species, ~7% of the dry weight is chitin and another 5% is the chitin derivative, chitosan^36,37^, so the ability to degrade these compounds would likely increase bacterial fitness in our microcosms. The chitin hydrolysis rate of the community supernatant was also highly variable across microcosms, with M03 and M09 having the highest measures of endochitinase activity (Fig. 4e). In summary, microcosm communities begin with different compositions as a result of historical contingencies affecting individual pitchers. These compositions shift as bacterial communities are brought into a new laboratory environment, but remain influenced by their starting compositions (Fig. 5, part i). We expected to see functional convergence (ii), since all communities were grown for many generations in the same environment. This convergence was observed in core functions, such as respiration, but not on chitinolytic activity or substrate utilization profiles. These “auxiliary” functions (encoded by transporters and enzymes at the periphery of the metabolic network) were instead strongly correlated with community composition, and thereby also affected by historical contingencies (iii).

We developed an isolate collection to learn more about the mechanisms underpinning the strong differences in substrate utilization and hydrolytic activity across microcosms. By testing the enzymatic activity and substrate metabolism of individual isolates, we hypothesized we could identify bacterial strains responsible for the corresponding community function. We successfully isolated 350 strains and sequenced their full 16S rRNA genes. Of these, 176 mapped with 100% identity to 33 different ASVs from the amplicon sequencing. For the five microcosms we cultured from, 14 out of the combined top 15 ASVs (in terms of relative abundance) on Day 63 matched perfectly with cultured strains, as did 5–7 of the top 10 ASVs per microcosm. Isolated strains accounted for 67-88% of each microcosm’s relative abundance on the final day of the experiment (M03: 88%; M05: 67%; M07: 69%; M09: 87%; and M10: 82%). Our cultured strains had broad representation across different taxonomic groups (Supplementary Data 1).

Consistent with our hypothesis, the chitinase activity of our cultured strains mirrored microcosm activity: strains from M03 and M09 had the highest endochitinase activity out of all measured strains. The activity of individual strains/ASVs mapped well to the activity of the entire microcosms, where M03 and M09 were also highest (Fig. 4e). In M03 only one of the measured strains showed high activity (ASV589, a *Chromobacterium* species), while in M09 at least three strains had high endochitinase activity (ASV018 *Chromobacterium*, ASV842 *Dyella*, and ASV863 *Burkholderia*). The cumulative endochitinase activity across strains (strain activity multiplied by corresponding ASV frequency over time) correlated with microcosm activity across all microcosms (linear model *R*^2^ = 0.2979, *p* < 0.0001), and within M03, M07 and M09 (*R*^2^ = 0.9303, *p* < 0.0001, *R*^2^ = 0.7044, *p* = 0.0029, and *R*^2^ = 0.5142, *p* = 0.0078, respectively. Supplementary Fig. 6). Interestingly, the strains with the highest chitinase activity also had high protease activity, and all except for ASV842 had high lipase activity (Supplementary Fig. 7), suggesting that these bacteria are generally specialized to degrade insect tissue.

To ask to what extent the pattern of substrate utilization of the community could be reduced to the substrate utilization of its members, we applied the same Biolog EcoPlates test to the individual isolates. We found that substrate utilization differences between communities could be attributed to the presence or absence of particular strains. Out of the 20 strains measured using Biolog EcoPlates, only one showed high metabolic activity when grown with salicylic acid (strain M09D5GC17 corresponding to ASV863 *Burkholderia*), and it was a strain present only in M09. The M09 community was the only one where growth on salicylic acid was observed. Conversely, the strain with the most growth on itaconic acid (strain M10D5GC19 corresponding to ASV140 *Achromobacter*) was present at the final timepoint in all the microcosms we cultured from except for M09, and the M09 community was the only one not able to grow on itaconic acid (Supplementary Fig. 8). Individual strains can thus drive functional differences among microcosms, and we were able to culture and analyze a set of these strains—connecting genotypes with their functional phenotypes.

## Discussion

The effects of historical contingencies are difficult to isolate and detect in the field, because even adjacent sites can experience distinct environmental conditions. The effects can be difficult to capture in a laboratory as well, because historical contingencies may only affect community assembly when disturbance is low and environmental selection is weak^2,12,38^ and these conditions are rarely met when natural communities are moved into laboratory settings. Our study demonstrates that historical contingency strongly influences community assembly in a realistic, but controlled, laboratory environment. Moreover, the effects of historical contingency are persistent and reproducible, suggesting that, with enough information about species’ functional capabilities, responses to environmental conditions, and interactions with other species, community assembly dynamics might be predictable.

We find the dynamics of individual ASVs affected key functional measures and were influenced by community context; likely driven by interactions among species within microcosms. For example, priority effects may have played a role, with species that quickly grow to high abundances altering growth conditions for other species. Microcosm communities remained different in richness and in composition, despite an initial shift when assembling in a constant in vitro environment. Surprisingly, strong differences also remained in terms of functional activity and functional dynamics were highly correlated with compositional dynamics in our study. Our results suggest that species across the microcosm communities were not functionally redundant with regard to chitinase activity, a relevant substrate degradation capability in the pitcher plant system. Redundancy in bacterial functional roles has been suggested as an explanation for the high complexity of microbial communities, and as a buffer that increases stability in the face of perturbation^35,39^. However, important but more “narrow” functional roles, such as the degradation of complex substrates, may often not be redundant. This study highlights how specific and relevant functional measures should be examined more closely in microbial ecology, because a general lack of redundancy in key functions could influence both the carbon flux and the overall stability of an ecosystem.

Our conceptual model for the assembly dynamics of microcosms suggests that when in the same in vitro environment, non-metabolically active species are quickly pruned, and after only one transfer the final diversity of the community is determined. This result implies that historical contingencies influence richness levels, even after communities equilibrate to a common environment. Despite biotic differences in richness and composition across microcosms we observe universal ASV extinction dynamics, as predicted by recent theory^34^. However, ASVs persisting within the microcosms are largely influenced by microcosm context, suggesting significant effects of biotic interactions or ecosystem engineering. Therefore, long-lasting effects of early conditions and biota lead to strong differences in final community composition and ecosystem function. The environmental conditions of our experiment supported multiple functional outcomes, which may have shifted the selective balance to species interactions, therefore increasing the possible community states. Our experiment was necessarily run in the laboratory, but it used wild communities as the starting point and suggests potential implications for a natural system. Stochastic events during colonization of the pitchers of carnivorous pitcher plants may have lasting impacts on the ability of the pitcher microbiome to degrade insect prey and to release nutrients, such as nitrogen and phosphorus, to the common pitcher pool in a plant-accessible form.

Our model system based on pitcher plant bacterial communities can be used to address other questions in microbial ecology. For example: the role of dispersal in community assembly; how coalescence events (the mixing of stable communities) lead to different compositions; how invasions alter community structure and function; and how evolution acts on individuals within communities to change interactions over time. Our microcosms are less complex than most natural systems because species that did not grow within the current environment were pruned during transfers, but are more complex than almost all experimental laboratory communities. The ability to culture key community members provides the opportunity for building synthetic communities that retain interactions among species previously established in nature.

## Methods

### Sample collection

We collected the entire aquatic pools from 10 healthy pitchers of *Sarracenia purpurea* pitcher plants at Harvard Pond (Harvard Forest, MA) in September, 2017. We used sterile, single-use pipettes to remove the samples into sterile 15 mL tubes. The samples were transported in a cooler on ice to the laboratory where they were refrigerated overnight. The following morning, we set up the experiment.

### Serial transfer experiment

We filtered half of each sample through 3 µm syringe filters to focus on the bacterial component of the community. From both the filtered and unfiltered components of each sample, we combined 500 µL of pitcher fluid with 500 µL of media in a 48-well plate. In order to have a complex nutrient source similar to what bacteria from pitcher plant fluids would experience in their native environment, we used un-buffered cricket media (3 g food-grade cricket powder from farmed *Acheta domestica* crickets purchased from Thailand Unique per 1 Liter of milliQ-purified water, acidified with HCl to pH 5.6 and then autoclaved). The plate was then placed in a 25 °C incubator. After three days of incubation, each sample was mixed well and 500 µL was transferred to a new plate with 500 µL of sterile cricket media. From our calculations, we added the equivalent of about 1/60^th^ of a cricket to every well at each transfer. We continued transferring samples and adding cricket media in a 1:1 ratio every three days for a total of 21 plates over 63 days.

At the beginning of the experiment (Day 0), we removed a portion of each sample to freeze at −80 °C for later DNA extraction and amplicon sequencing. We removed 100 µL of each filtered sample to first measure optical density (OD) at 600 nm, and then used a Fluorimetric Chitinase Assay Kit (Sigma-Aldrich) to measure the activity of three different types of chitinases: endochitinases, chitobiosidases and β-N-acetylglucosaminidases. We bead-beat each sample for 1 min and centrifuged it before using a portion of the supernatant in the assay, with two replicates for each sample. Our downstream analyses focused on endochitinases, the enzymes that cleave intramolecular bonds forming new chain ends.

We also measured a “functional fingerprint” of the communities with Biolog EcoPlates^40^. EcoPlates measure the metabolism of 31 carbon sources (listed in Supplementary Fig. 5). Microbes respire when utilizing a carbon source, and this reduces a tetrazolium redox dye to a visible purple color. Water is used as a control to measure any background respiration, which is then subtracted from the other responses. We diluted each sample 1:10, combining 1 mL of each filtered sample with 9 mL of purified water that had been acidified to pH 5.6 and then autoclaved. We filled each EcoPlate well with 100 μL of sample, and incubated the plates in the 25 °C incubator for three days. At the end of this time the plate was read in a plate reader to quantify the tetrazolium dye response by measuring OD at 590 nm.

In addition to measuring chitinase and EcoPlate activity, we measured CO_2_ production with the MicroResp system^41^. The MicroResp system uses a cresol red indicator dye in agar as a detector medium above a deepwell plate, which can be filled with the relevant substrate and microbial communities. We added 250 µL of each filtered sample to 250 µL of cricket media in three replicates for each sample in a deepwell plate, attached a detector plate with the MicroResp seal and clamp, and incubated at 25 °C for three days before measuring the resulting color change in the detector plate at 570 nm. We calibrated the MicroResp measurements according to the manual by incubating the detector medium with known CO_2_ concentrations and making a reference curve.

At each transfer, we repeated the MicroResp measurement and froze a portion of the culture at −80 °C for later DNA extraction. Every second transfer, we repeated the chitinase activity measurements, and every third transfer we repeated the Biolog EcoPlates with a 1:40 dilution to reduce carry over of any remaining cricket medium. All measurements after the first sets were done without replicates. During the course of the experiment, some of the MicroResp indicator plates showed evidence of fungal contamination; measurements involving affected wells were removed from our analyses.

On Day 15 of the serial transfer experiment, we removed 100 μL of fluid from each microcosm and examined it under a microscope. We recorded a binary score of protozoan activity as visible or not visible. No protozoan activity was visible in any of the filtered microcosms, but there was activity in the unfiltered samples of M02, M05, and M06, indicating that protozoan predation persisted longest in these communities.

On the final day (Day 63) of the experiment, we repeated all functional measurements, cultured five of the ten microcosm communities in order to isolate strains, and froze 100 µL of each community in 40% glycerol solution and the remaining culture at −80 °C.

### DNA extraction and sequencing

DNA was extracted from all samples with the Agencourt DNAdvance kit (Beckman Coulter) using 100 µL per sample and overnight lysis at 55 °C with rapid shaking (300 rpm). In each 96-well extraction plate we included negative controls. DNA was quantified with the Quant-iT PicoGreen dsDNA Assay kit (Invitrogen) on a plate reader, before being sent to the Environmental Sample Preparation and Sequencing Facility at Argonne National Laboratory for amplicon sequencing on a MiSeq targeting the V4 region of 16S rRNA using the 515F and 806 R primers^42,43^. Each 25 µL PCR reaction contained 9.5 µL of MO BIO PCR Water (Certified DNA-Free), 12.5 µL of QuantaBio’s AccuStart II PCR ToughMix (2x concentration, 1x final), 1 µL Golay barcode tagged Forward Primer (5 µM concentration, 200 pM final), 1 µL Reverse Primer (5 µM concentration, 200 pM final), and 1 µL of template DNA. The conditions for PCR were as follows: 94 °C for 3 min to denature the DNA, with 35 cycles at 94 °C for 45 s, 50 °C for 60 s, and 72 °C for 90 s; with a final extension of 10 min at 72 °C to ensure complete amplification. Amplicons were then quantified using PicoGreen (Invitrogen) and a plate reader (Infinite® 200 PRO, Tecan) before being pooled in equimolar amounts. This pool was then cleaned with AMPure XP Beads (Beckman Coulter), and quantified using a fluorometer (Qubit, Invitrogen). After quantification, the molarity of the pool was determined and it was diluted down to 2 nM, denatured, and then diluted to a final concentration of 6.75 pM with a 10% PhiX spike for sequencing on the Illumina MiSeq. Amplicons were sequenced on a 151 bp × 12 bp × 151 bp MiSeq run using customized sequencing primers and procedures^42^.

### Amplicon sequence analysis

On the MIT Engaging computing cluster, we used QIIME2 version 2018.4^44^ to demultiplex our sequences, and the DADA2^45^ plugin to denoise sequences and to generate Amplicon Sequence Variants (ASVs) of ~250 base pairs in length. We retained all ASVs with more than two sequences across all samples. We assigned taxonomy using the classify-sklearn method which is a Naive Bayes classifier, and a pre-trained classifier made with the Greengenes database, version 13_8. The phylogenetic tree was built using the QIIME2 SEPP^46^ plugin.

Statistical analyses were performed and graphs were generated in R and Mathematica. Reads for each ASV were normalized by the total amount of reads in each sample. Bray–Curtis dissimilarities and NMDS ordinations were performed using the R *vegan* package^47^. Weighted and unweighted UniFrac distances were calculated using the R *phyloseq* package^48^. NMDS ordinations were run in two dimensions (*k* = 2) using the metaMDS command in *vegan* and stress levels were reported on the figures. The effective number of species was calculated as exp(Shannon index). Richness for Fig. 2 was calculated counting all ASVs present at later time points in each microcosm as present at previous time points to account for ASVs below the sequencing detection limit. Richness of early timepoints (Days 0 and 3) were correlated with that of the final timepoint (Day 63) using linear regression. We used additional R packages, including *picante*, *plyr*, *ggplot2*, *reshape2*, *dendextend*, and *biclust*.

### Null model for community assembly

To describe the dynamics of ASV loss, we employed a geometric model, i.e., the probability *P*(*t*) of an ASV that goes extinct eventually to go extinct at transfer *t* is equal to the probability that it did not go extinct in the preceding *t* − 1 transfer. That is,

$$P\left( t \right) = p\left( {1 - p} \right)^{t \, - \, 1},$$

Where *p* is the sole parameter of the model. It can be shown that the maximum likelihood estimator of *p* is given by the mean time to extinction, i.e., $\hat p = 1/t$. To determine whether the extinction time distributions are best described by a single parameter *p* or whether individual parameters for each microcosm are needed, we computed the Bayesian Information Criterion *B* from the likelihood ${\cal{L}}$ of the observed extinction under the geometric model, using either a single parameter *p* given by the inverse of the mean extinction time for all ASVs across all microcosms, or for each microcosm individually, i.e.,

$${\cal{B}}_k = \ln(n)k - {\cal{L}}\left( {{\mathrm{data|}}\left\{ p \right\}_k} \right),$$

where *k* is either 1 (for a common parameter) or 10 (for individual parameters) and *n *= 10 microcosms. We found ${\cal{B}}_1 = 2237,\,{\cal{B}}_{10} = 2187,$ such that the relative likelihood of the common-parameter model over the individual-parameter model was $e^{({\cal{B}}_1 - {\cal{B}}_{10})/2} \approx 7.7\times 10^{10}$.

### Correlation analysis

For the correlation analysis in Fig. 3d, ASV abundances were first center-log transformed after removal of ASVs that were never observed in a given microcosm. One pseudo-read was added for all other ASVs to account for ASV abundances below the detection threshold. Standard Pearson correlation coefficients were then computed using the transformed time series. The same procedure was applied after first aggregating ASVs by family for Supplementary Fig. 4. We computed the correlation coefficients of time series of the same ASV in different microcosms (from the same or different inoculum) and compared them to correlations between randomly chosen ASVs in different microcosms. For Fig. 3e, we measured the similarity in composition between communities by the Bray–Curtis distance on the untransformed relative abundance and the similarity between ASV dynamics by the cosine similarity between untransformed relative abundance time series. The cosine similarity metric uses the cosine of the angle between vectors and measures similarity irrespective of size. It was chosen in order to automatically remove time points where the ASV was not observed in one or both microcosms.

### Null model for an ASV having the same fate in multiple microcosms

For Supplementary Fig. 3b, we classified the fate (either extinction of persistence) of individual ASVs shared between microcosms. For the null model, we begin with each strain at a 50% (random) chance of having the same fate in a new microcosms as it had in its current microcosm, and estimate the probability *P(n)* of a similar strain occurring in *n* microcosms to have to same fate in all *n* microcosms as $P(n) = f^{n - 1}$, which is shown as the red line in Supplementary Fig. 3b.

### Strain isolation and identification

Individual strains were isolated from five of the ten microcosms (M03, M05, M07, M09, and M10) by plating the culture fluid and picking around 100 colonies per microcosm. We cultured on petri plates at 1:10^5^ and 1:10^6^ dilutions using both cricket media with the addition of agar and a second medium containing 11.28 g/L M9 salts, vitamin solution^49^, trace metals^50^, agar, and 2.5 g/L *N*-acetylglucosamine (GlcNAc) as the sole carbon source. Plates were put in a 25 °C incubator for 1 week, after which single colonies were picked and then re-streaked at least two additional times before being grown up in liquid media and frozen in glycerol at −80 °C.

To identify and barcode our strains, we added 2 µL of liquid culture to 20 µL of sterile, nuclease free water and, after a freeze-thaw cycle, did direct PCR using primers 27F and 1492R to amplify the 16S ribosomal RNA gene and the Q5 High-Fidelity kit (New England Biolabs) with an initial 5-min incubation at 100 °C. Before Sanger sequencing, we tested for successful amplification with gel electrophoresis and cleaned the PCR products with SPRI beads according to the Agencourt AMPure XP protocol. Sanger sequences were trimmed and filtered with Geneious, and assigned taxonomy using the RDP classifier.

### Measurements of strain functional activity

We measured the functional activity of ~50 strains with 100% matches in their Sanger-sequenced 16S rRNA gene to ASVs from the amplicon sequencing. When multiple strains mapped to the same ASV, their enzyme activities were averaged. Each strain was streaked out on cricket-M9 media plates from the frozen glycerol stock, and then a single colony was grown in liquid cricket media. Chitinase activity was measured as described for the microcosm communities, and for Fig. 4e strains were considered to be active above a cutoff of 1 unit/mL. Protease and lipase activities were also measured using the Sigma-Aldrich Protease Fluorescent Detection Kit and Lipase Activity Assay Kit III. Seventeen of the strains were put into EcoPlates to compare their metabolic activity on the 31 substrates to that of their source communities.

### Reporting summary

Further information on research design is available in the Nature Research Reporting Summary linked to this article.

## Supplementary information

Supplementary Information

Peer Review File

Reporting Summary

Description of Additional Supplementary Files

Supplementary Data 1

## Data Availability

Sequence data have been deposited in the NCBI Sequence Read Archive (SRA) under Project ID PRJNA559886. Any other relevant material is available from the corresponding author upon request.

## Source Data

Source Data

## Code availability

R and Mathematica code and source data tables for all of our figures are available via the Harvard Dataverse: 10.7910/DVN/U3QJQZ.
