# Supplementary material for: Context-dependent dynamics lead to the assembly of functionally distinct microbial communities
Source: Nat Commun. 2020 Mar 18;11:1440. doi: 10.1038/s41467-020-15169-0 (PMC7080782; doi:10.1038/s41467-020-15169-0)
Supplement: Supplementary file 3 — Reporting Summary [file 41467_2020_15169_MOESM3_ESM.pdf]

nature research

Corresponding author(s): Otto X. Cordero

Last updated by author(s): 2/13/2020

Reporting Summary

Nature Research wishes to improve the reproducibility of the work that we publish. This form provides structure for consistency and transparency in reporting. For further information on Nature Research policies, see Authors & Referees and the Editorial Policy Checklist .

Statistics

For all statistical analyses, confirm that the following items are present in the figure legend, table legend, main text, or Methods section.

n/a	Confirmed	
☐	☒	The exact sample size (*n*) for each experimental group/condition, given as a discrete number and unit of measurement
☐	☒	A statement on whether measurements were taken from distinct samples or whether the same sample was measured repeatedly
☐	☒	The statistical test(s) used AND whether they are one- or two-sided<br>*Only common tests should be described solely by name; describe more complex techniques in the Methods section.*
☒	☐	A description of all covariates tested
☐	☒	A description of any assumptions or corrections, such as tests of normality and adjustment for multiple comparisons
☐	☒	A full description of the statistical parameters including central tendency (e.g. means) or other basic estimates (e.g. regression coefficient) AND variation (e.g. standard deviation) or associated estimates of uncertainty (e.g. confidence intervals)
☐	☒	For null hypothesis testing, the test statistic (e.g. *F*, *t*, *r*) with confidence intervals, effect sizes, degrees of freedom and *P* value noted<br>*Give P values as exact values whenever suitable.*
☒	☐	For Bayesian analysis, information on the choice of priors and Markov chain Monte Carlo settings
☒	☐	For hierarchical and complex designs, identification of the appropriate level for tests and full reporting of outcomes
☐	☒	Estimates of effect sizes (e.g. Cohen's *d*, Pearson's *r*), indicating how they were calculated

Our web collection on statistics for biologists contains articles on many of the points above.

Software and code

Policy information about availability of computer code

Data collection	No software was used for data collection.
Data analysis	Data in this study was analyzed using: QIIME2 version 2018.4, DADA2, Mathematica version 11.3, R version 3.5.1, and R packages: vegan 2.5-2, ggplot2 3.5.2, dendextend 1.9.0, reshape2 3.5.1, biclust 3.5.2, ggtern 3.0.0, phyloseq 2013, and picante 3.5.2. Mathematica and R code, along with the relevant data tables, are accessible from the Harvard Dataverse: https://doi.org/10.7910/DVN/U3QJQZ.

For manuscripts utilizing custom algorithms or software that are central to the research but not yet described in published literature, software must be made available to editors/reviewers. We strongly encourage code deposition in a community repository (e.g. GitHub). See the Nature Research guidelines for submitting code & software for further information.

Data

Policy information about availability of data

All manuscripts must include a data availability statement. This statement should provide the following information, where applicable:

- Accession codes, unique identifiers, or web links for publicly available datasets
- A list of figures that have associated raw data
- A description of any restrictions on data availability

Sequence data has been deposited in the NCBI Sequence Read Archive (SRA) under Project ID PRJNA559886. R code and data tables are available via the Harvard Dataverse: https://doi.org/10.7910/DVN/U3QJQZ.

Field-specific reporting

Please select the one below that is the best fit for your research. If you are not sure, read the appropriate sections before making your selection.

Ecological, evolutionary & environmental sciences study design

All studies must disclose on these points even when the disclosure is negative.

Study description	The aquatic communities from 10 individual Sarracenia purpurea pitchers were inoculated into a realistic, complex nutrient source: sterilized, ground crickets in acidified water. The in vitro communities from both filtered and unfiltered inocula were serially transferred every three days for 21 transfers, using a low dilution rate of one-part culture to one-part fresh media. Community composition was measured for each transfer using 16S rRNA sequencing. Ecosystem function was measured in the filtered communities in three ways: 1) CO2 production at every transfer, 2) chitinase production at every second transfer, and 3) substrate utilization across 31 substrates at every third transfer.
Research sample	We collected all the fluid present from 10 pitchers of Sarracenia purpurea, the purple pitcher plant. Pitcher plants were used for this experiment because each one contains a small ecosystem, with a unique assembly history. We targeted pitchers that were healthy (no evidence of herbivory or senescence) and contained sufficient fluid to set up our experiment. The sample represents the metacommunity present across many pitchers in Harvard Pond.
Sampling strategy	We chose a sample size of 10 original pitchers as this number generated a feasible quantity of samples (430) for amplicon sequencing when collecting both filtered and unfiltered samples every 3 days over the course of our 2-month experiment.
Data collection	L.S.B collected the original samples, set up the experiment, and collected experimental samples for amplicon sequencing as well as measurements of CO2 production via the MicroResp system, chitinase production via a Sigma kit, and substrate utilization via the EcoPlate system.
Timing and spatial scale	The original samples were collected on September 18, 2017 from Harvard Pond (Harvard Forest, MA). The spatial scale of collections was about 100 meters, a distance across which bacteria can easily disperse. We chose to collect samples at the end of the summer when pitchers were still healthy and active, and microbiomes had assembled. Harvard Forest is a key research site for studies of Sarracenia purpurea pitcher plants.
Data exclusions	Some of our MicroResp indicator plates showed evidence of fungal contamination during the experiment, and measurements involving affected wells were removed from our analyses.
Reproducibility	We did the experiment with both filtered and unfiltered pitcher fluids, and the unfiltered samples are like a repetition of the experiment under slightly different conditions. The communities from both types of fluids followed very similar trajectories in terms of composition and structure (see Supplementary Figure S2), indicating that our results are reproducible.
Randomization	Randomization was not relevant for this study, as we did not assign experimental groups.
Blinding	Blinding was not relevant for this study, as we did not have experimental groups.

Did the study involve field work? ☒ Yes ☐ No

Field work, collection and transport

Field conditions	Samples were collected on a partially sunny day with temperatures between 16-21 deg. C and no rainfall.
Location	Samples were collected from Harvard Pond, part of Harvard Forest in Petersham, MA. Latitude 42.501712, Longitude -72.208440, and elevation 340 meters.
Access and import/export	All samples were legally collected with permission from Harvard Forest. No samples were imported to or exported from the USA.
Disturbance	Disturbance was minimized by using a canoe for sample collection and not stepping on fragile habitats. Collection of pitcher fluid is non-destructive and does not harm the plants.

Reporting for specific materials, systems and methods

We require information from authors about some types of materials, experimental systems and methods used in many studies. Here, indicate whether each material, system or method listed is relevant to your study. If you are not sure if a list item applies to your research, read the appropriate section before selecting a response.

Materials & experimental systems

n/a	Involved in the study
☒	☐ Antibodies
☒	☐ Eukaryotic cell lines
☒	☐ Palaeontology
☒	☐ Animals and other organisms
☒	☐ Human research participants
☒	☐ Clinical data

Methods

n/a	Involved in the study
☒	☐ ChIP-seq
☒	☐ Flow cytometry
☒	☐ MRI-based neuroimaging
