# Supplementary material for: Context-dependent dynamics lead to the assembly of functionally distinct microbial communities
Source: Nat Commun. 2020 Mar 18;11:1440. doi: 10.1038/s41467-020-15169-0 (PMC7080782; doi:10.1038/s41467-020-15169-0)
Supplement: Supplementary file 5 — Supplementary Data 1 [file 41467_2020_15169_MOESM5_ESM.pdf]

Supplementary Information for:

Context-dependent dynamics lead to the assembly of functionally distinct microbial communities

Bittleston, et al 2020

Supplementary Figures

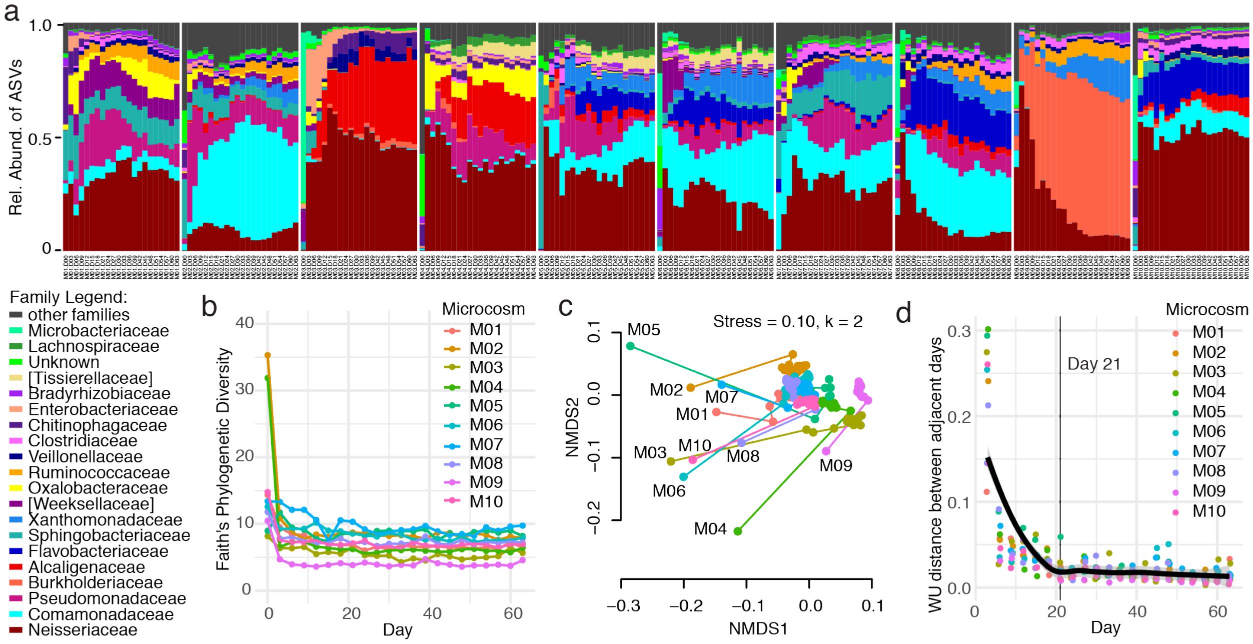

Supplementary Figure 1. Phylogenetic comparisons across microcosms. a) Relative abundance at the family level in each microcosm over time. b) Faith's phylogenetic diversity of each microcosm over time. c) NMDS plot as in Figure 1b but using the weighted Unifrac metric. d) The change in weighted Unifrac distance in microcosms over time.

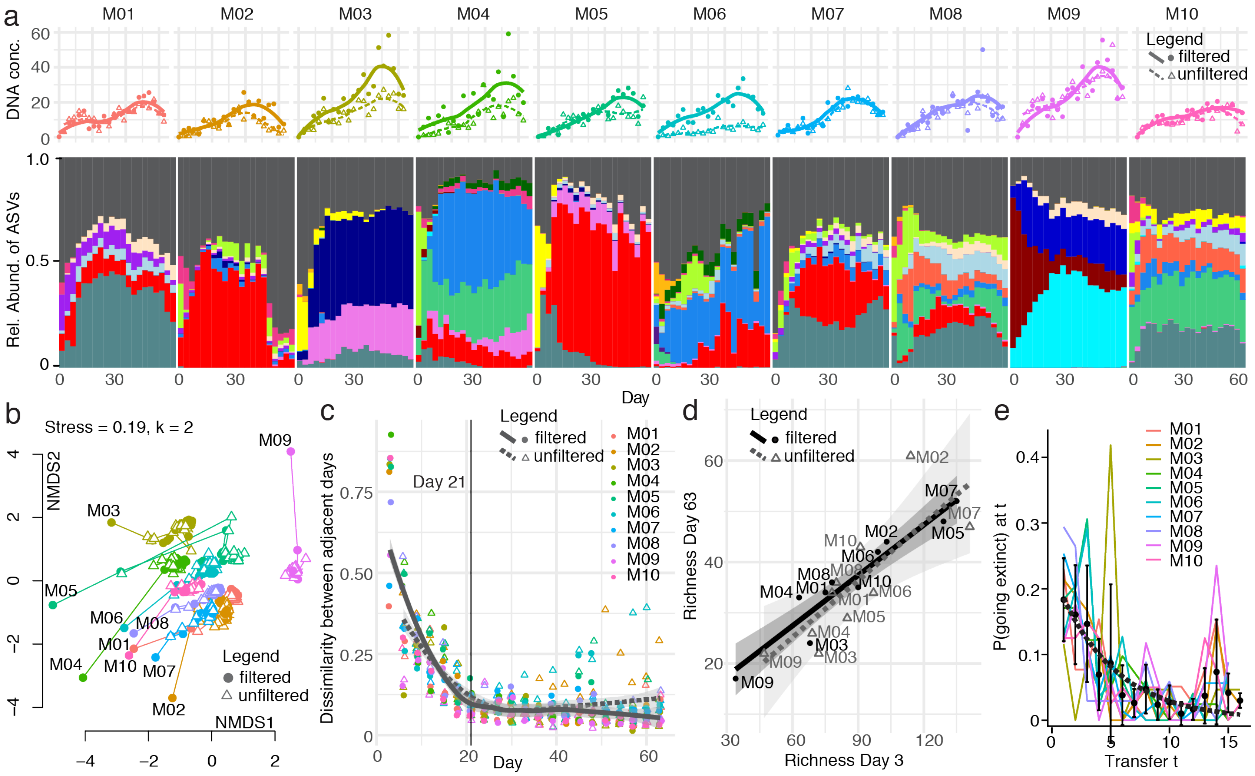

Supplementary Figure 2. Effects of environmental selection and historical contingency are replicated in both filtered and unfiltered microcosm communities. a) The bar plot shows relative abundances of the same 20 ASVs displayed in Figure 1a from the unfiltered communities. Above the bar plot, DNA concentrations are displayed showing filtered (solid circles and solid line) and unfiltered (open triangles and dashed line) samples over time. b) Filtered (solid circles) and unfiltered (open triangles) samples follow almost the same trajectories, and are very similar in the NMDS ordination of their Bray-Curtis dissimilarities over time. Note that unfiltered communities were not sequenced for Day 0. c) The change in Bray-Curtis dissimilarity of communities between adjacent days over time is similar in filtered (solid circles and solid line) and unfiltered (open triangles and dashed line) communities. d) Richness on Day 3 and Day 63 are correlated in both filtered (solid circles and solid line) and unfiltered (open triangles and dashed line) communities. Linear model for unfiltered: $R^2 = 0.585$, $p = 0.006$. e) Plot as in Figure 2c but for unfiltered samples. The probability of going extinct at transfer *t*. Colored lines are probability densities for individual microcosms. Black points are averages across microcosms, the black line is the maximum likelihood distribution with a common parameter across microcosms (see main text and Methods) given by the inverse mean extinction time.

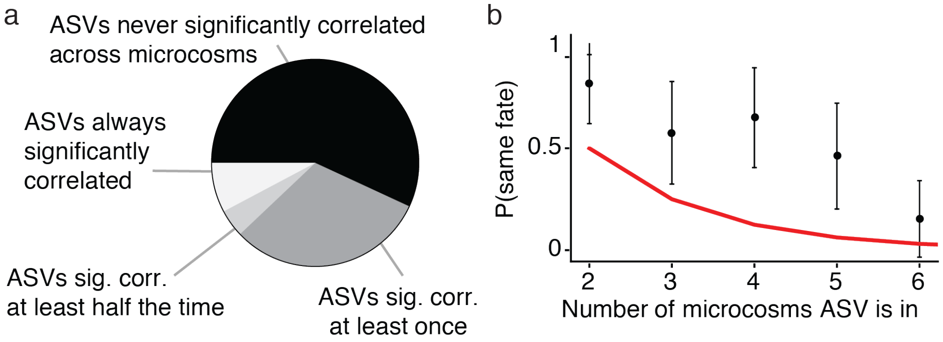

Supplementary Figure 3. Correlations of ASV dynamics in different microcosms. a) Pie chart showing proportions of ASVs that are never, at least once, at least half of the time, or always significantly correlated in different microcosms. b) Probability of ASVs having the same fate (either persisting or going extinct) in different microcosms depending on the number of microcosms they are present in. Red line shows null expectation (see Methods).

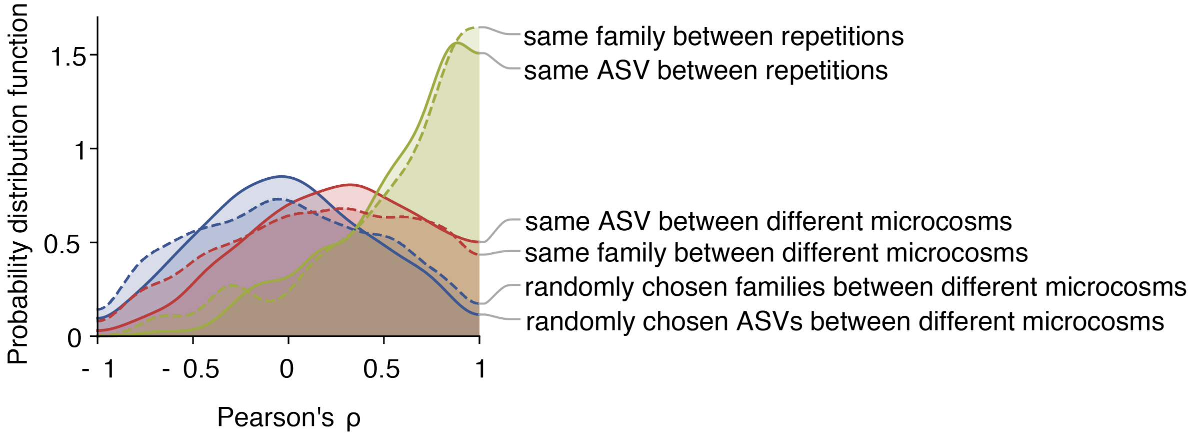

Supplementary Figure 4. Similar correlations are found when comparing ASVs or bacterial families between repetitions and across microcosms, suggesting that family is almost as informative as ASV. As in Figure 3d, we show the probability density function of correlation coefficients between the same families as compared with ASVs in repetitions started from the same inocula, in microcosms started from distinct initial communities, and between randomly chosen ASVs.

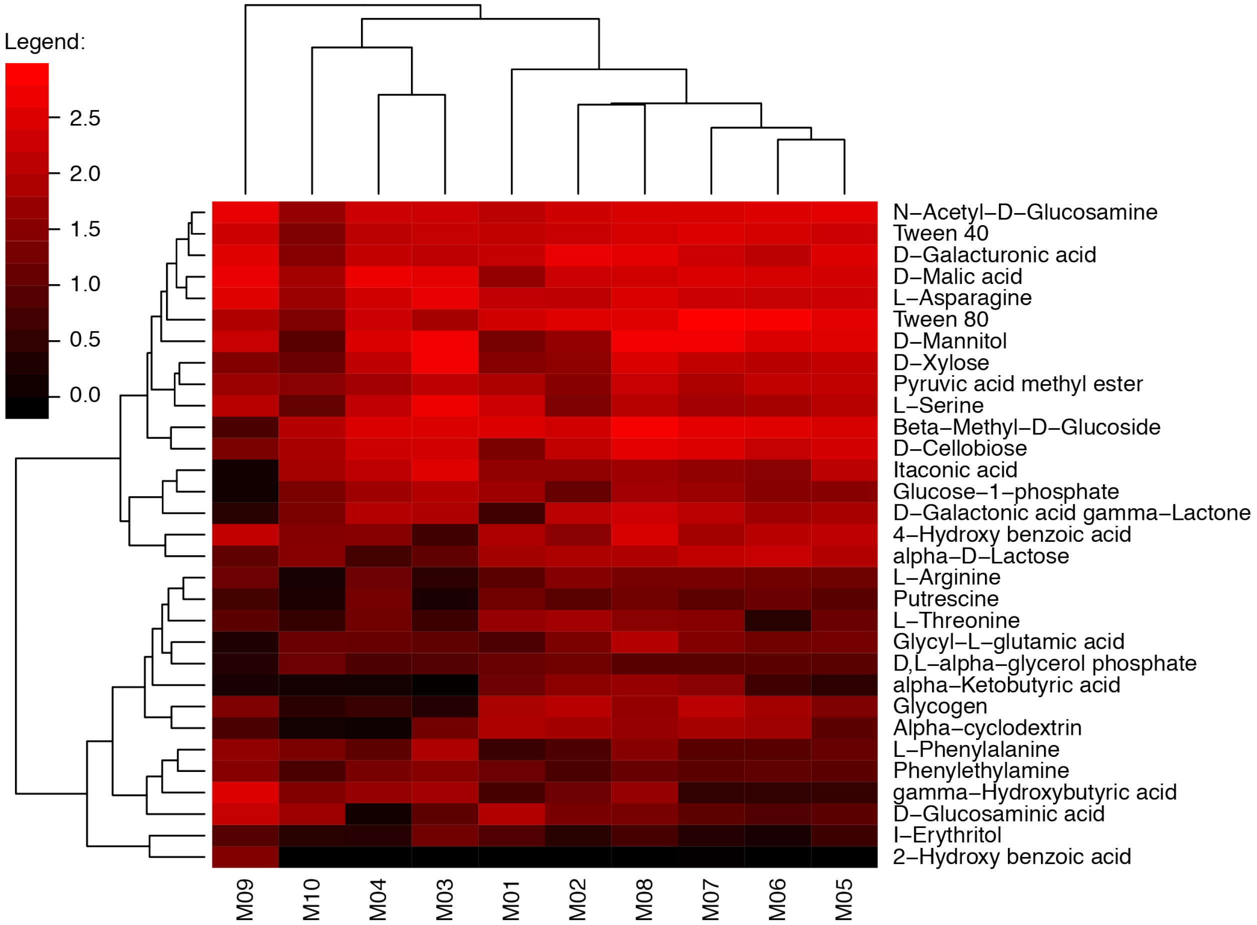

Supplementary Figure 5. Heatmap of EcoPlate data for stabilized microcosms (averaged across Day 27 to Day 63). Microcosms (columns) and substrates (rows) are arranged by similarity according to hierarchical clustering.

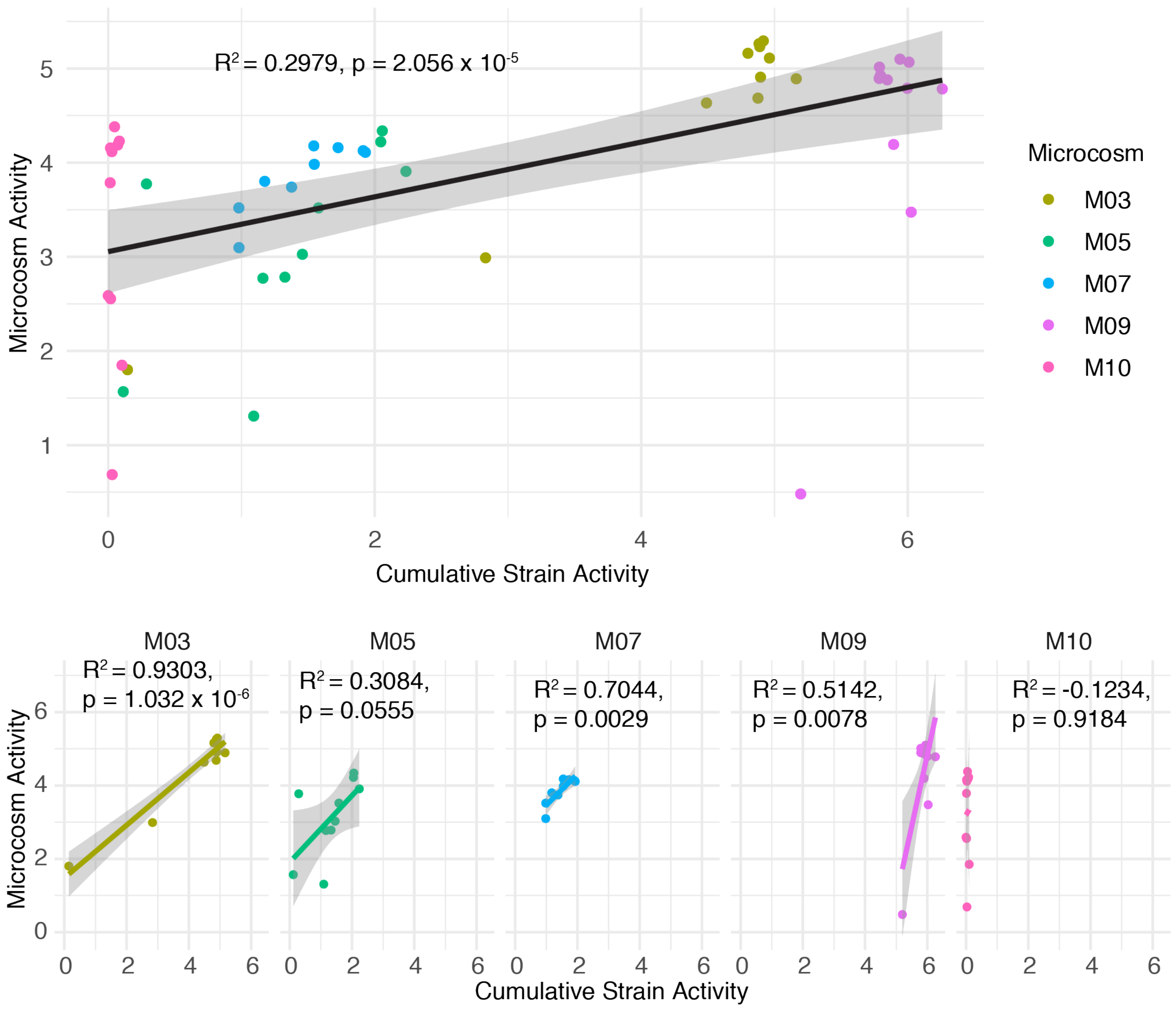

Supplementary Figure 6. Linear models indicate that strain endochitinase activity correlates with microcosm endochitinase activity, both across all microcosms, and within M03, M07 and M09. The natural logarithm of the enzyme activity was used, to better capture the broad spread of the activities. Endochitinase activity was measured using a Fluorimetric Chitinase Assay Kit as described in the methods.

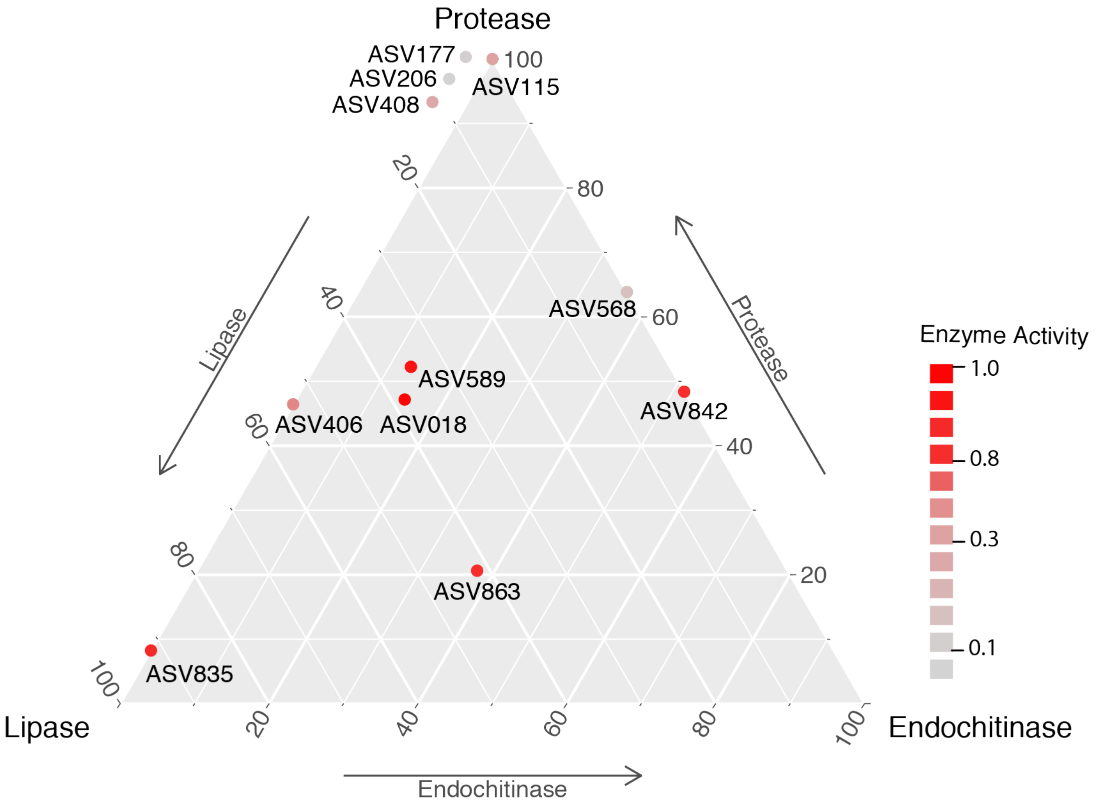

Supplementary Figure 7. Ternary plot of enzyme activity (endochitinase, lipase and protease) in cultured strains, mapped to corresponding ASV. Enzyme activity was normalized across strains and only values about 0.04 are included in this plot. The color legend for enzyme activity reflects the maximum value across all three enzymes. Strains with high chitinase activity generally also have high protease and lipase activity (positioned near the center of the plot).

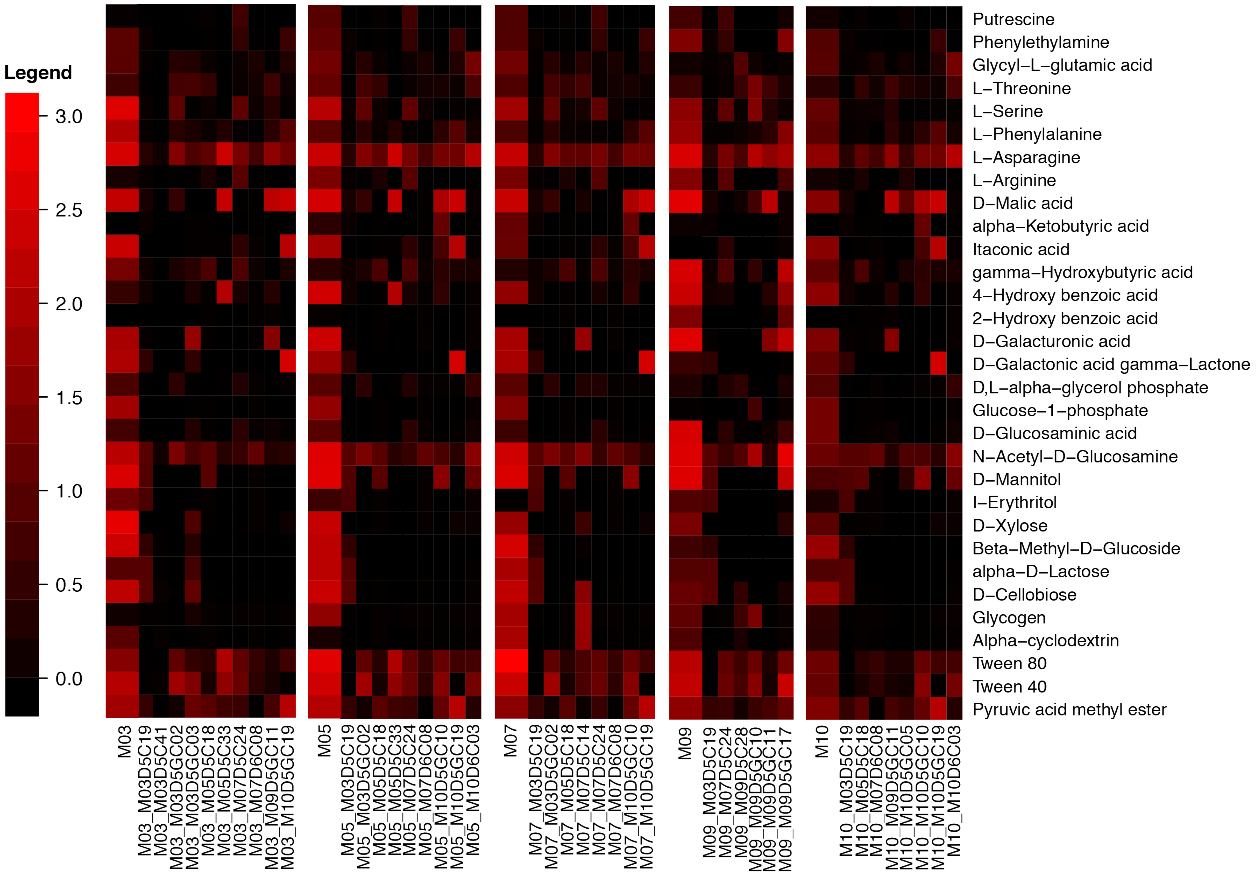

Supplementary Figure 8. Heatmap of EcoPlate results for Day 63 of Microcosms where strains were cultured, as well as a subset of the cultured strains (organized by which were present in each microcosm). Rows show EcoPlate substrates, columns are either communities or strains. The legend shows how color corresponds to substrate use: black is substrate not used, red is high use.
