# Supplementary material for: Context-dependent dynamics lead to the assembly of functionally distinct microbial communities
Source: Nat Commun. 2020 Mar 18;11:1440. doi: 10.1038/s41467-020-15169-0 (PMC7080782; doi:10.1038/s41467-020-15169-0)
Supplement: Supplementary file 4 — Description of Additional Supplementary Files [file 41467_2020_15169_MOESM4_ESM.pdf]

Reviewers' Comments:

Reviewer #1:
Remarks to the Author:
The article ¨Context-dependent dynamics lead to the assembly of functionally distinct pitcher-plant microbiomes¨ (NCOMMS-19-27510-T) asks an important question in the field of microbial ecology: to what extent and how does historical contingency processes in community assembly affect also the final function of the community, in addition to the composition? To address this question, the authors use a system that allows to elegantly manipulate initial conditions by using communities from different naturally occurring pools as a starting point. Then, they stabilize these communities in a rich synthetic m edium and look at convergence in final composition and function.

Overall, I think the authors have done a great deal of work to address the questions, and the result is a potentially very interesting work. However, I do believe also that as it is now, the article has important issues that need to be addressed before publication, which are listed below.

--

The first claim of the paper is that communities assemble in different states due to historical contingency. Historical contingency can be defined as a process in which initially, a random variation in species composition generates idiosyncratic assembly conditions - built environments, or niche construction. These newly generated conditions, in turn, select for different dynamics in each species and result eventually in different final compositional states. Results show that a) compositionally different starting points result in different endpoints, b) there common patterns regarding global dynamics of diversity and extinction, c) there is a strong influence of the initial richness on the final one, and d) the dynamics of the same isolates in different microcosms are different. I do believe these points show significant evidence that we are looking at a historical contingency process; however, I do have several important concerns:

1. A more careful evaluation of the NMSD analysis is needed, e.g. a methods section and at least reporting the stress value. This would apply to all NMDS uses throughout the paper.

2. The fact that many of the high abundance strains are shared by many microcosms is essential for the paper. In my opinion, the figure S1 is a bit involved and tricky to read (no legend, confusing axes annotation), and I'd like to see it summarized in a more clear way in the main text. One suggestion is a simple panel showing the distribution of the number of microcosms a strain appears in, but authors are of course free to decide on the best way to present their data.

3. I think that context-dependent differences in species dynamics, which is one of the main claims of the paper, should be characterized in more quantitative detail. If the species dynamics depend on the context, we would expect to see that the dynamics (or at least the final abundance) of a species are more similar in microcosms where that species has a similar context. Coarse-graining to the order or family level might be useful for this analysis.

4. It would be nice to see whether there is compositional convergence between microcosms at higher taxonomic levels, despite diversity at lower taxonomic levels. In particular, it seems that Neisseriales and Burkholderiales would recurrently make the bulk of the microcosms (I might be wrong on this as my impression comes only from visual inspection of Fig. 1a). It might be useful to see a plot

equivalent to Fig. 1A but colored by higher taxonomic level (e.g. order, family). However, this is not essential, I leave it as a minor point for the authors to decide whether to address it.

5. I struggle to understand why is the additional experiment without filtration important. Given that technically it is not a replicate, what should we expect? This result seems to show that predation is not important for the assembly (which does not seem particularly relevant), and suggests that community assembly is reproducible from the same pool. But if the authors want to prove that assembly and final composition is reproducible when starting from the same pool, that would require proper replicates from each pool. Such replicates are necessary also to state that contingency arises from the compositional variation in the initial pools, and not from e.g. sampling errors or bottlenecks when inoculating.

The second part of the paper addresses whether the assembled communities are functionally different, and measures several types of metabolic function, from respiration to the use of several substrates, including chitin. The authors present compelling evidence that the assembled communities are not only different in composition, but also in function. I also have several problems with this second, function-related part.

6. The authors do describe in the text how well the isolated strains represent the communities (L238-241), but I'm missing a more clear graphical analysis. How much of the total abundance of the five microcosms where strains have been isolated corresponds to the isolated strains?

7. Authors offer qualitative evidence that chitinase activity in communities broadly maps chitinase activity in their isolates. I would suggest going one more step and trying to quantitatively predict the function of the overall community with the sum of the functions of the isolates. Due to the presence of non-isolated strains in the communities, it could be hard to disentangle how much of the unexplained function is due to interactions versus non-isolated strains. For example, besides interactions in which strains affect each other's growth, they could be also stimulating each other's production of chitinase. But it seems to me that for 3 out of the five analyzed communities, the isolates don't predict the whole community chitinase activity (fig. 4). I believe that at least an attempt to do this analysis more quantitatively and a discussion of the results could significantly improve the paper.

8. The paper shows that while some broad functions are convergent (respiration) other, more narrow functions are not, and depend to some extent on the composition of the final community. But in the introduction, the authors recognize (L64-75) that functional convergence will be highly dependent on the actual function that we are focusing on. Thus, from the authors' own logic, the results they obtain later on are not really surprising. In addition, the conceptual model laid out in figure 5 will be different depending on the function considered. I would ask the authors to discuss the more general implications of their results, and how are their conclusions important beyond the particular system and functions used in this work.

Reviewer #2:
Remarks to the Author:
This paper by Bittleston et al. describes an experiment whereby microbial community assembly was studied using in vitro microcosms seeded by natural communities derived from pitcher plants. The authors find that unique stable communities states were reached by 21 days of culture, and that the structure and characteristics of these communities converged, or were similar in some respects, but that each microcosm also maintained the unique features that likely derived from the differences in their starting inocula.

Overall, I found this paper to be an interesting read – the experiment was quite ambitious and the amount of data generated was substantial and allowed for a detailed analysis and characterization of the system. The paper is clearly written for the most part, and the results are well presented. I think this work could become an important contribution in the field of microbial ecology, providing insight into how microbial communities change over time, particularly from a functional perspective. However, there are a few things I hope the authors could address, which may help improve the paper.

Detailed comments:

Considering the authors' argument regarding the importance of historical contingency in shaping microbiomes, and the implication that this effect could be almost deterministic, I was surprised that there were no strict replicates conducted for any of the 10 treatments. Even having one or two replicate samples would have greatly helped support (or refute) some of the ideas presented in the paper. I think this is the greatest weaknesses of the paper. However, they do note that the same experiment was performed with unfiltered inocula (line 188), and the results seem to support the idea that trajectories of the microbial communities are reproducible (although this is not a strict replicate). I think this should be emphasized more, and there should be some discussion on this. Do the same ASV's win out in the same proportions in the unfiltered experiment? Do these results support the authors' richness and extinction models in Lines 123-163?

Line 72-77: good points, I agree!

Line 132: very interesting observation regarding richness. Has this been seen before in other studies? Line 134-136, this could be simple case of culture bias: do the authors know how closely their artificial media replicates the conditions in a pitcher plant?

On that point, it is also important to compare the communities in their artificial microcosms with the natural communities they originated from, since this essentially constrains what functions each microcosm has to draw upon. It would be good to have some information on diversity metrics, particularly phylogenetic diversity (a loose proxy for functional diversity), comparing say day 63 vs day 0. Generally, I am also curious why Unifrac metric was not used to examine beta diversity in addition to Bray-Curtis, and why there is no commentary regarding the similarities and differences of microcosms with related strains versus more distantly related strains. I think accounting for phylogeny is necessary when making comparisons and interpreting the results, since ASVs are not independent from one another, but also carry the historical baggage of their evolutionary history.

Line 173-180: this part is very hard to follow and I'm not sure what the point is. I suggest restructuring to make it clearer. For one, the time series correlation discussed here could be explained better.

Figure 3c: I wonder if the same effect will be observed if comparing ASV's of similar function or phylogeny (e.g. genera) between different microcosms?

Figure 3e: regarding the null expectation, the method described to generate this seems a bit circular to me. Would it not also make sense to include a line showing the expectation if the fate of the strains were uncorrelated? E.g., assuming the 50% extinction rate reported in Line 160. I also suggest making it clear what the "null" is in the figure legend, without having to refer to the Methods.

Line 191: what is this "activity" and how was it observed/measured?

Figure 4b: clearly state the time "direction" of the plotted points (I assume the points beside the labels are day 0?)

Line 231 and Figure 5: I agree historical contingencies are key factors here. However, the whole premise of functional convergence seems to really depend on what one considers a "function" and whether different starting communities even have the capability of convergence in the first place. Do the authors think the lack of convergence is due to differences in the starting communities regarding encoded potential functions? Or is it due to subsequent biotic interactions? Both of these hypotheses might be difficult to test, although one can do metagenomic sequencing to perhaps check the former. Regarding the idea that functional differences are maintained, this is perhaps not surprising given the relatively short timespan of the study. If the authors were to continue passaging for a million generations, they will see more similarities as genes not under selection will get lost, and beneficial genes may be gained or up-regulated! This brings me to the question of how much the functional differences measured by the authors are indeed relevant to the system – ie., how strongly are these functions selected for in their growth conditions? How important is metabolism of salicylic acid and itaconic acid and chitin, etc. for fitness? How do the authors know they are actually measuring the most important "functions"? They can say that there are many functional differences between their microcosms, but isn't that to be expected if these functions are essentially neutral in this particular system? These comments are not criticisms per se, but I suggest the authors discuss these points, and the limitations, a little more in the paper.

Line 237: what were these other strains that did not map to ASV's? Where did they come from? Are they contaminants?

Line 279-280: how could "early colonizers" be defined in these microcosms, when all strains are added simultaneously at the start of the experiment?

Line 296-297: regarding ASV extinction dynamics, what do the authors mean exactly when they suggest that it is driven by "external factors in the transfer process"? Are they suggesting that the transfers are removing dead/inactive cells at a certain rate, leading to similar-appearing extinction patterns across microcosms?

Line 298-302: the authors imply significant effect of species interactions, yet there is no evidence in their results to support this. For instance, they do not show any metabolic complementarity between strains, or evidence of cooperation, conflict, syntrophy, or how the growth medium is altered by certain strains. This is fine; a study doesn't need to test everything, but it does seem a bit hand-wavy to say that species interactions are "significant" effects that explain the observations, when this was not examined explicitly.

Lines 331-332: more details are needed regarding the culture media. How was the cricket powder made, exactly? What acid was used to acidify the media? Was the media buffered, and if so, with what?

Line 370: does the DNA extraction method include bead-beating? Some gram-positive cells could be missed if only relying on chemical methods of DNA extraction.

Supplementary Data Table 1: 16S sequences and/or percentage hits to known strains should be given for each cultured strain.

Figure S4: what do the colors for enzyme activity indicate? Average across all three enzymes, or for endochitinase only? It is not clear.

Title: is it still fair to call the microcosms in this paper "pitcher-plant microbiomes", since by the end of the experiment, they are quite different from the day 0 communities (Fig. 1a)? The title might be misleading; despite the origins of the starting inocula, the study is actually describing an artificial system.

Reviewer #3:
Remarks to the Author:
The study presents a targeted assessment to determine the effect of historical contingency for the establishment and functioning of microbial communities. This is a fundamental question with highest relevance for predicting and managing the dynamics and functional capabilities of microbiota for human purposes. The starter communities are transferred from a natural (pitcher plant) to a synthetic microcosm environment and a serial transfer experiment is conducted and modelled to specify the impact of composition, function and context-dependency on final communities. They find that historical contingency significantly impacts final composition and diversity, that composition drives function and functional redundancy is less relevant in the pitcher plant system, because the compositional context that likely enables the establishment of specific interactions, determines microbial productivity. The experimental and computational set-up is very elegant as it directly aims for resolving the posed questions without further complications. The arguments are well selected and convincing. Some aspects however, remain slightly unassertive, which I therefore point out further in my comments.

Comments

Results 178-184/Figure 3e What would be the expectation for the fates in individual microcosms in a random ASV context without adaptation knowing about the geometric extinction dynamics you describe earlier? Please support your conclusion for context-dependency with a statistical model/test. E.g. add a line to the plot for expected fates in the random case. The same test for any two ASVs pairs could also be informative and directly setting ASVs in context suggesting ecological interactions - as opposed to only comparing to the background.
Results 183 The currently presented results do not address the topic of interactions. Please reformulate or tone down the final statement.

Results 217-220/Figure 4d Please explain in more detail the analysis and the plot. I understand you are comparing distances of richness between subsequent time points versus distances of function-readout between subsequent time points. I don't see how this supports the conclusion that similar composition implements similar function? Do you link function read-out to composition beyond procrusts? As is I would say the change dynamics (=distances) is correlated.
The punctuation in the labels of plot 4d are easily misunderstood for divided by, please adapt accordingly.

Results 230-232/Fig 5 '..functional differences correlated with their composition' As above, either show same-function-same-composition directly or change to e.g. '…functional dynamics correlate with compositional dynamics over time'.

Minor

Introduction 87 A clear naming of the experiment type is advisable (Serial transfer experiment).

Results 164 Explain more clearly what the expectations are for investigating individual ASVs after knowing that extinction is driven by the serial transfer condition. E.g. ASVs that behave significantly different due to adaptive functions/interactions.

Results 242 I suggest to clearly state that chitinase activity is the relevant function for the original plant context to point out the host-microbiome function trade off and underline the essentiality as microcosm readout.
Results 253 ..substrate usage sum of strain usage.. Please present numbers or statistical test to support this statement.

Discussion 275, 299 'interactions'. Please indicate in the text that interaction statements are likely assumptions.
Methods 347 Please explain which readouts are available/chosen in the EcoPlate assay.

Reviewers' comments:

Reviewer #1 (Remarks to the Author):

The article ¨Context-dependent dynamics lead to the assembly of functionally distinct pitcher-plant microbiomes¨ (NCOMMS-19-27510-T) asks an important question in the field of microbial ecology: to what extent and how does historical contingency processes in community assembly affect also the final function of the community, in addition to the composition? To address this question, the authors use a system that allows them to elegantly manipulate initial conditions by using communities from different naturally occurring pools as a starting point. Then, they stabilize these communities in a rich synthetic medium and look at convergence in final composition and function.

Overall, I think the authors have done a great deal of work to address the questions, and the result is a potentially very interesting work. However, I do believe also that as it is now, the article has important issues that need to be addressed before publication, which are listed below.

We thank the reviewer for the positive assessment. Below is a point-by-point response to the reviewer's concerns.
--

The first claim of the paper is that communities assemble in different states due to historical contingency. Historical contingency can be defined as a process in which initially, a random variation in species composition generates idiosyncratic assembly conditions - built environments, or niche construction. These newly generated conditions, in turn, select for different dynamics in each species and result eventually in different final compositional states. Results show that a) compositionally different starting points result in different endpoints, b) there common patterns regarding global dynamics of diversity and extinction, c) there is a strong influence of the initial richness on the final one, and d) the dynamics of the same isolates in different microcosms are different. I do believe these points show significant evidence that we are looking at a historical contingency process; however, I do have several important concerns:

A more careful evaluation of the NMSD analysis is needed, e.g. a methods section and at least reporting the stress value. This would apply to all NMDS uses throughout the paper.

We have included more information about the NMDS analysis in the methods, and have added number of dimensions and stress values to each NMDS plot.

The fact that many of the high abundance strains are shared by many microcosms is essential for the paper. In my opinion, the figure S1 is a bit involved and tricky to read (no legend, confusing axes annotation), and I'd like to see it summarized in a more clear way in the main text. One suggestion is a simple panel showing the distribution of the

number of microcosms a strain appears in, but authors are of course free to decide on the best way to present their data.

We appreciate this suggestion and have included a new panel (d) in Figure 1 that addresses proportion and relative abundances of ASVs in different numbers of microcosms. We also summarize it in the main text in lines 129-132.

I think that context-dependent differences in species dynamics, which is one of the main claims of the paper, should be characterized in more quantitative detail. If the species dynamics depend on the context, we would expect to see that the dynamics (or at least the final abundance) of a species are more similar in microcosms where that species has a similar context. Coarse-graining to the order or family level might be useful for this analysis.

This is a great suggestion and we have added two new panels (d and e) to Figure 3, illustrating this key point. Panel 3d shows how ASV's are more correlated in repetitions (filtered and unfiltered samples) from the same inoculum than in different microcosms. This analysis was coarse-grained to the family level in Supplementary Figure S4, which shows the same pattern. Panel 3e shows how the cosine distance (essentially, the correlation) between trajectories of the same ASV in different microcosms increases with increasing Bray-Curtis dissimilarity between final community compositions. This shows explicitly that ASV dynamics depend on community context.

It would be nice to see whether there is compositional convergence between microcosms at higher taxonomic levels, despite diversity at lower taxonomic levels. In particular, it seems that Neisseriales and Burkholderiales would recurrently make the bulk of the microcosms (I might be wrong on this as my impression comes only from visual inspection of Fig. 1a). It might be useful to see a plot equivalent to Fig. 1A but colored by higher taxonomic level (e.g. order, family). However, this is not essential, I leave it as a minor point for the authors to decide whether to address it.

We have added a supplementary figure S1 with a bar plot of our samples at the family level. While many families are shared, certain microcosms remain very distinct, even at the family level.

I struggle to understand why is the additional experiment without filtration important. Given that technically it is not a replicate, what should we expect? This result seems to show that predation is not important for the assembly (which does not seem particularly relevant), and suggests that community assembly is reproducible from the same pool. But if the authors want to prove that assembly and final composition is reproducible when starting from the same pool, that would require proper replicates from each pool. Such replicates are necessary also to state that contingency arises from the compositional variation in the initial pools, and not from e.g. sampling errors or bottlenecks when inoculating.

This is an important point. Although, technically speaking we do not have replicates for each of the ten microcosms, unfiltered samples mirror the corresponding filtered ones. Our previous analysis did not show this fact properly, and that is one of the main corrections we have made to the new manuscript. Our argument is thus that, even though unfiltered samples were treated differently at t=0, the fact that their dynamics and composition are highly correlated with the corresponding filtered microcosms indicates that community assembly dynamics are deterministic. Therefore, the differences observed across microcosms are due to the differences in the initial species pool and the fact that taxa behave in a context dependent manner. We have included additional analyses and figures to highlight the relevance of the unfiltered samples for our study. Supplementary Figure S2 illustrates how the unfiltered samples follow the same dynamics and can act as effective replicates in this study. Furthermore, figure 3c shows a strong correlation in UniFrac distances between filtered and unfiltered communities. Relevant edits can be found in lines 151-163 and 203-224.

The second part of the paper addresses whether the assembled communities are functionally different, and measures several types of metabolic function, from respiration to the use of several substrates, including chitin. The authors present compelling evidence that the assembled communities are not only different in composition, but also in function. I also have several problems with this second, function-related part.

The authors do describe in the text how well the isolated strains represent the communities (L238-241), but I'm missing a more clear graphical analysis. How much of the total abundance of the five microcosms where strains have been isolated corresponds to the isolated strains?

The cultured strains account for 67-88% of the relative abundance on the final day of the experiment, depending on the microcosm. We have included this information in more detail in lines 277-279.

Authors offer qualitative evidence that chitinase activity in communities broadly maps chitinase activity in their isolates. I would suggest going one more step and trying to quantitatively predict the function of the overall community with the sum of the functions of the isolates. Due to the presence of non-isolated strains in the communities, it could be hard to disentangle how much of the unexplained function is due to interactions versus non-isolated strains. For example, besides interactions in which strains affect each other's growth, they could be also stimulating each other's production of chitinase. But it seems to me that for 3 out of the five analyzed communities, the isolates don't predict the whole community chitinase activity (fig. 4). I believe that at least an attempt to do this analysis more quantitatively and a discussion of the results could significantly improve the paper.

For reasons stated by the reviewer, this is hard to do, but we have added more direct quantitative analysis in Supplementary Figure S6. We do find a significant correlation when comparing the endochitinase activity of cultured strains multiplied by their frequencies over time to that of the whole microcosm. We also find significant

correlations between chitinolytic strain abundance and microcosm activity over time for microcosms M03, M07 and M09. Relevant edits can be found in lines 287-291.

The paper shows that while some broad functions are convergent (respiration) other, more narrow functions are not, and depend to some extent on the composition of the final community. But in the introduction, the authors recognize (L64-75) that functional convergence will be highly dependent on the actual function that we are focusing on. Thus, from the authors' own logic, the results they obtain later on are not really surprising. In addition, the conceptual model laid out in figure 5 will be different depending on the function considered. I would ask the authors to discuss the more general implications of their results, and how are their conclusions important beyond the particular system and functions used in this work.

This is an interesting point. Based on our results, our argument is not only that functional convergence depends on context, but that this dependence is predictable: core functions, such as respiration (or choice of terminal electron acceptor, in general) are likely to be relatively independent of taxonomic composition. By contrast, auxiliary functions (peripheral with respect to the structure of the metabolic network) are likely to be directly linked to taxonomic composition. The reason is that those functions on the periphery of the network (e.g. transport and hydrolysis) evolve faster and can be highly variable even between strains of the same species. The functions measured by EcoPlates recover profiles of substrate utilization which directly depend on these auxiliary functions. This argument is explained in the discussion of our revised paper, Lines 265-269.

Reviewer #2 (Remarks to the Author):

This paper by Bittleston et al. describes an experiment whereby microbial community assembly was studied using in vitro microcosms seeded by natural communities derived from pitcher plants. The authors find that unique stable communities states were reached by 21 days of culture, and that the structure and characteristics of these communities converged, or were similar in some respects, but that each microcosm also maintained the unique features that likely derived from the differences in their starting inocula.

Overall, I found this paper to be an interesting read – the experiment was quite ambitious and the amount of data generated was substantial and allowed for a detailed analysis and characterization of the system. The paper is clearly written for the most part, and the results are well presented. I think this work could become an important contribution in the field of microbial ecology, providing insight into how microbial communities change over time, particularly from a functional perspective. However, there are a few things I hope the authors could address, which may help improve the paper.

We thank the reviewer for the positive comments. Below we address each comment in detail.

Detailed comments:

Considering the authors' argument regarding the importance of historical contingency in shaping microbiomes, and the implication that this effect could be almost deterministic, I was surprised that there were no strict replicates conducted for any of the 10 treatments. Even having one or two replicate samples would have greatly helped support (or refute) some of the ideas presented in the paper. I think this is the greatest weaknesses of the paper. However, they do note that the same experiment was performed with unfiltered inocula (line 188), and the results seem to support the idea that trajectories of the microbial communities are reproducible (although this is not a strict replicate). I think this should be emphasized more, and there should be some discussion on this. Do the same ASV's win out in the same proportions in the unfiltered experiment? Do these results support the authors' richness and extinction models in Lines 123-163?

We appreciate the reviewer's suggestion to better incorporate the unfiltered inocula, and have included additional analyses and more discussion of these samples. Our analysis shows that, even though these samples were treated differently at t=0, their dynamics and composition mirror that of the filtered microcosms. Supplementary Figure 2 illustrates how the unfiltered samples follow essentially the same dynamics. Furthermore, figure 3c shows comparison of filtered and unfiltered samples based on UniFrac distances and corroborates that the unfiltered samples behave as effective replicates. We have also utilized the unfiltered samples in new analyses to better show the context-dependency of ASV activity (in Figure 3d and e). Overall, these analyses show that community dynamics are deterministic, and that the different states reached by the microcosms are determined by the initial inocula, in agreement with the notion of historical contingencies.

Line 72-77: good points, I agree!

Thank you!

Line 132: very interesting observation regarding richness. Has this been seen before in other studies?

We have not found other experimental examples of this in the literature, but we did find the same result predicted in a theory paper from Dr. Stefano Allesina's lab, which has now been added as a reference and described in Lines 190-193.

Line 134-136, this could be simple case of culture bias: do the authors know how closely their artificial media replicates the conditions in a pitcher plant?

Sarracenia purpurea pitcher plant fluid has not been extensively analyzed, but it is known that the plant produces only a very small amount of the fluid, and the vast majority is made up of rainwater, insect prey, and living organisms. It is also known that their fluid is acidic (pH ~ 5). We have attempted to match these conditions by using insects as the food source and acidifying the media.

On that point, it is also important to compare the communities in their artificial microcosms with the natural communities they originated from, since this essentially constrains what functions each microcosm has to draw upon. It would be good to have some information on diversity metrics, particularly phylogenetic diversity (a loose proxy for functional diversity), comparing say day 63 vs day 0. Generally, I am also curious why Unifrac metric was not used to examine beta diversity in addition to Bray-Curtis, and why there is no commentary regarding the similarities and differences of microcosms with related strains versus more distantly related strains. I think accounting for phylogeny is necessary when making comparisons and interpreting the results, since ASVs are not independent from one another, but also carry the historical baggage of their evolutionary history.

We agree that evolutionary history is important, and we have added a comparison of phylogenetic diversity over time in Supplemental Figure S1, panel b. Day 0 generally has higher phylogenetic diversity, which largely decreases by Day 3. Unfortunately, it is impossible to distinguish whether these ASVs were already dead / metabolically inactive in the pitcher plants or whether they were unable to grow in our experimental conditions. We chose to primarily use Bray-Curtis dissimilarities in the main text of the paper, so that they were directly comparable with the EcoPlate functional analyses which are also done with Bray-Curtis. However, we recognize that phylogenetic measures can provide more information and so we have included NMDS plots with weighted UniFrac distances in Supplementary Figure S2. We also added a new plot using unweighted UniFrac in Figure 3c.

Line 173-180: this part is very hard to follow and I'm not sure what the point is. I suggest restructuring to make it clearer. For one, the time series correlation discussed here could be explained better.

We have completely re-written this section (now lines 207-224) and have added new panels, Figures 3d and 3e, illustrating how ASV dynamics depend on community context.

Figure 3c: I wonder if the same effect will be observed if comparing ASV's of similar function or phylogeny (e.g. genera) between different microcosms?

To address this, we ran the analysis shown in the current Figure 3d panel at the family level as well. We have added this as a new Supplementary Figure S4. The results are very similar, indicating that ASV and family provide almost the same information for this particular analysis.

Figure 3e: regarding the null expectation, the method described to generate this seems a bit circular to me. Would it not also make sense to include a line showing the expectation if the fate of the strains were uncorrelated? E.g., assuming the 50% extinction rate reported in Line 160. I also suggest making it clear what the "null" is in the figure legend, without having to refer to the Methods.

We agree that it makes more sense to begin with a 50% extinction rate, and have revised the null expectation and the figure accordingly. Due to the addition of new panels in Figure 3 that are more informative, we have moved this figure to the supplemental material, Supplementary Figure S3.

Line 191: what is this "activity" and how was it observed/measured?

We have added more information to the methods about the protozoan activity and how it was observed (lines 412-416).

Figure 4b: clearly state the time "direction" of the plotted points (I assume the points beside the labels are day 0?)

Thank you for noticing this omission, we have added the information to the figure legend.

Line 231 and Figure 5: I agree historical contingencies are key factors here. However, the whole premise of functional convergence seems to really depend on what one considers a "function" and whether different starting communities even have the capability of convergence in the first place. Do the authors think the lack of convergence is due to differences in the starting communities regarding encoded potential functions? Or is it due to subsequent biotic interactions? Both of these hypotheses might be difficult to test, although one can do metagenomic sequencing to perhaps check the former. Regarding the idea that functional differences are maintained, this is perhaps not surprising given the relatively short timespan of the study. If the authors were to continue passaging for a million generations, they will see more similarities as genes not under selection will get lost, and beneficial genes may be gained or up-regulated!

It is correct that whether functional convergence is observed depends on what function one considers. We show that core functions, such as respiration, are rather independent of composition, whereas auxiliary (or "peripheral") functions like substrate utilization are much more dependent on species composition. This is consistent with the notion that genes for substrate uptake are much more variable between organisms, even closely related ones. With this in mind, we think the functional differences are due to differences in species composition. We also make the point that differences in composition after stabilization are driven by historical contingencies that lead to differential ASV dynamics. The fact that ASV dynamics change as a function of community context (Fig 3d) suggests that that biotic interactions (e.g. via niche construction) change species dynamics and therefore indirectly change substrate

utilization profiles. The fact that the function of the isolates is consistent with the function of the community (analysis of chitinase activity, Figure 4 and new Supplementary Figure S6) is consistent with this notion. Finally, we agree that the maintenance of the function is not surprising, but we did not intend to present it as a surprising result, simply as an observation that corroborates the idea that communities have come close to an equilibrium. We find it difficult to speculate about what would happen after a million generations. Depending on the shape of the fitness landscape evolutionary dynamics need not be ergodic and can be also trapped in alternative states for long time. These musings, however, are tangential to our results.

This brings me to the question of how much the functional differences measured by the authors are indeed relevant to the systm – ie., how strongly are these functions selected for in their growth conditions? How important is metabolism of salicylic acid and itaconic acid and chitin, etc. for fitness? How do the authors know they are actually measuring the most important "functions"? They can say that there are many functional differences between their microcosms, but isn't that to be expected if these functions are essentially neutral in this particular system? These comments are not criticisms per se, but I suggest the authors discuss these points, and the limitations, a little more in the paper.

This is an interesting point. Chitin is particularly relevant to the system because it is the main component of insect exoskeletons. This relevant function is also highly variable and correlated with composition, which shows that despite strong selection microcosms don't converge on relevant functions. We have elaborated more on this in lines 265-269. With respect to the EcoPlates, we use these as a generic functional fingerprint of the communities, indicating functional differences that likely go beyond the 31 carbon compounds in the assay (this point added, lines 242-244).

Line 237: what were these other strains that did not map to ASV's? Where did they come from? Are they contaminants?

Some of the other strains mapped to ASVs at less than 100% (e.g. 20 strains were greater than 97% but less than 100%). The rest are most likely a combination of low-quality sanger sequences, mixed cultures, and strains below detection limit in the sequencing but that grow well on plates.

Line 279-280: how could "early colonizers" be defined in these microcosms, when all strains are added simultaneously at the start of the experiment?

We meant species that grow early vs. those that peak late. We have changed our language to "species that quickly grow to high abundances." Lines 322-323.

Line 296-297: regarding ASV extinction dynamics, what do the authors mean exactly when they suggest that it is driven by "external factors in the transfer process"? Are they suggesting that the transfers are removing dead/inactive cells at a certain rate, leading to similar-appearing extinction patterns across microcosms?

We realized this sentence was sloppy and potentially confusing. We have removed that phrase from the revised version. The extinction dynamics are a manifestation of the selection process and the difference in extinction dynamics for the same ASV result from the fact that taxa dynamics are context dependent.

Line 298-302: the authors imply significant effect of species interactions, yet there is no evidence in their results to support this. For instance, they do not show any metabolic complementarity between strains, or evidence of cooperation, conflict, syntrophy, or how the growth medium is altered by certain strains. This is fine; a study doesn't need to test everything, but it does seem a bit hand-wavy to say that species interactions are "significant" effects that explain the observations, when this was not examined explicitly.

Here, we disagree. We show in the revised version that species dynamics change gradually as community composition diverges (Fig 3d) and that dynamics are reproducible (comparisons with unfiltered samples). What can explain the differences in dynamics when the only variable that is different is community context? It seems reasonable to expect that biotic effects drive these differences. Now, biotic effects do not need to imply a direct pairwise interaction, but could also be niche construction via changes in the pH or oxygen or the metabolite pool. We revised the language and also added more information about why we believe species interactions are acting here ("Because community composition is the only factor that changes across microcosms, this result implies that biological interactions (e.g. competition, niche construction via secreted metabolites, etc.) are the main drivers of population dynamics" Lines 222-224.

Lines 331-332: more details are needed regarding the culture media. How was the cricket powder made, exactly? What acid was used to acidify the media? Was the media buffered, and if so, with what?

We have added in more detail in the methods about where we purchased the cricket powder, how we used HCl to acidify the media, and that it was not buffered. This media is designed to mimic pitcher plant fluid. Lines 376-378.

Line 370: does the DNA extraction method include bead-beating? Some gram-positive cells could be missed if only relying on chemical methods of DNA extraction.

We did not include bead-beating in the DNA extraction method, but did use overnight lysis with rapid shaking (information added, Line 423). It is possible that some gram-positive cells may not have been lysed; however, we see the same gram-positive taxonomic groups in our current study as found in past studies of Sarracenia purpurea microbes where bead-beating was used in the DNA extractions (e.g. Bittleston et al. 2018 eLife).

Supplementary Data Table 1: 16S sequences and/or percentage hits to known strains should be given for each cultured strain.

16S sequences and top BLAST hit to a named genus have been added to the supplementary file.

Figure S4: what do the colors for enzyme activity indicate? Average across all three enzymes, or for endochitinase only? It is not clear.

Thank you for bringing this up, we have added that information to the legend. The color represents the maximum enzyme activity across the three enzymes.

Title: is it still fair to call the microcosms in this paper “pitcher-plant microbiomes”, since by the end of the experiment, they are quite different from the day 0 communities (Fig. 1a)? The title might be misleading; despite the origins of the starting inocula, the study is actually describing an artificial system.

To avoid confusion, we have removed “pitcher-plant microbiomes” from the title and replaced it with “microbial communities.”

Reviewer #3 (Remarks to the Author):

The study presents a targeted assessment to determine the effect of historical contingency for the establishment and functioning of microbial communities. This is a fundamental question with highest relevance for predicting and managing the dynamics and functional capabilities of microbiota for human purposes. The starter communities are transferred from a natural (pitcher plant) to a synthetic microcosm environment and a serial transfer experiment is conducted and modelled to specify the impact of composition, function and context-dependency on final communities. They find that historical contingency significantly impacts final composition and diversity, that composition drives function and functional redundancy is less relevant in the pitcher plant system, because the compositional context that likely enables the establishment of specific interactions, determines microbial productivity. The experimental and computational set-up is very elegant as it directly aims for resolving
the posed questions without further complications. The arguments are well selected and convincing. Some aspects however, remain slightly unassertive, which I therefore point out further in my comments.

We thank the reviewer for the positive comments. Below we address each comment in detail.

Comments

Results 178-184/Figure 3e What would be the expectation for the fates in individual microcosms in a random ASV context without adaptation knowing about the geometric extinction dynamics you describe earlier? Please support your conclusion for context-dependency with a statistical model/test. E.g. add a line to the plot for expected fates in the random case. The same test for any two ASVs pairs could also be informative and

directly setting ASVs in context suggesting ecological interactions - as opposed to only comparing to the background.

Thank you for pointing this out, another reviewer noted it as well. We have revised the null expectation to begin with a 50% (random) chance of extinction. Due to the addition of new analyses and new panels in Figure 3 that are more informative, we have moved panels d and e to the supplemental material, Supplementary Figure S3.

Results 183 The currently presented results do not address the topic of interactions. Please reformulate or tone down the final statement.

We have changed our language to better describe our reasoning here. Lines 222-224.

Results 217-220/Figure 4d Please explain in more detail the analysis and the plot. I understand you are comparing distances of richness between subsequent time points versus distances of function-readout between subsequent time points. I don't see how this supports the conclusion that similar composition implements similar function? Do you link function read-out to composition beyond procrusts? As is I would say the change dynamics (=distances) is correlated.

We compare dissimilarities in composition between all timepoints across all microcosms against the dissimilarities in functional profile for the corresponding samples. We show that if two samples are similar in composition, they are also similar in function. The figure shows that function and composition are tightly linked. We have updated our explanation in the figure legend.

The punctuation in the labels of plot 4d are easily misunderstood for divided by, please adapt accordingly.

We have changed the labels for plot 4d to fix this issue.

Results 230-232/Fig 5 '..functional differences correlated with their composition' As above, either show same-function-same-composition directly or change to e.g. '…functional dynamics correlate with compositional dynamics over time'.

See our previous response.

Minor

Introduction 87 A clear naming of the experiment type is advisable (Serial transfer experiment).

We have changed the text as suggested.

Results 164 Explain more clearly what the expectations are for investigating individual

ASVs after knowing that extinction is driven by the serial transfer condition. E.g. ASVs that behave significantly different due to adaptive functions/interactions.

We have revised our language to be clearer (lines 189 and 194-195). Extinction rates within each microcosm followed a universal pattern, but the fate of a particular ASV was different depending on which microcosm it was in. This implies that species dynamics are dependent on the biotic context defined at early timepoints, with consequences for function (Figure 4).

Results 242 I suggest to clearly state that chitinase activity is the relevant function for the original plant context to point out the host-microbiome function trade off and underline the essentiality as microcosm readout.

Thank you, this is a good suggestion and we have included more information about chitin and its relationship to the pitcher plant system in the text. Lines 84-87 and 256-259.

Results 253 ..substrate usage sum of strain usage.. Please present numbers or statistical test to support this statement.

We have changed the language here to be more precise, and have added new analyses correlating chitinase activity in the strains with that of the microcosms, Supplementary Figure S6 and Lines 287-291 and 297.

Discussion 275, 299 ‘interactions’. Please indicate in the text that interaction statements are likely assumptions.

We changed the language at what used to be line 299 to: “ASVs persisting within the microcosms are largely influenced by microcosm context, suggesting significant effects of species interactions or ecosystem engineering” (Lines 342-343).

Methods 347 Please explain which readouts are available/chosen in the EcoPlate assay.

We have added more detail about the EcoPlate assay analysis in the methods, Lines 395-397.

Reviewers' Comments:

Reviewer #1:
Remarks to the Author:
I thank the authors for their detailed response and the additional analyses done. They addressed my questions and concerns in a satisfactory manner. I believe the additions significantly strengthen the main conclusions that historical contingency and context-dependent dynamics can have profound consequences on the function of microbial communities. I thus recommend the article for publication

Reviewer #2:
Remarks to the Author:
The authors have done a good job of addressing the concerns of the reviewers. The revised paper is much improved, with many of the previously confusing components clarified, and being more focused and better supported with the evidence presented, overall. I have a few more comments:

Line 113: Regarding using DNA concentration as a proxy for biomass, I'm wondering if this was verified with more standard microbiological methods (e.g. OD measurement, CFU counts).

Line 213: Regarding "marginally higher" statement, the differences (Line 214) seem large to me.

Line 220-221: Please explain in more detail this new analysis. "Cosine similarity in ASV dynamics" is not very intuitive.

Line 489-495: This seems unchanged from the first submission. Fig. 3e is different now.

Figure 3d: "Pick". Is this a typo?

Figure S2a: in the relative abundance graph, is this showing the filtered or unfiltered samples, or combined?

Figure S6: Please explain what/how this activity was measured/calculated. E.g., which enzymes were used?

Reviewer #3:
Remarks to the Author:
Thank you for your work on the manuscript. I find it much improved and my comments suitably addressed.
With one exception 394-401: Please explicitely state what the functional fingerprint of the Biolog EcoPlates actually means/measures (e.g. different C sources). Explain, what the dye response represents and how this differs from the MicroResp system.

REVIEWERS' COMMENTS:

Reviewer #1 (Remarks to the Author):

I thank the authors for their detailed response and the additional analyses done. They addressed my questions and concerns in a satisfactory manner. I believe the additions significantly strengthen the main conclusions that historical contingency and context-dependent dynamics can have profound consequences on the function of microbial communities. I thus recommend the article for publication

Thank you for your comments, we are glad we could address your concerns.

Reviewer #2 (Remarks to the Author):

The authors have done a good job of addressing the concerns of the reviewers. The revised paper is much improved, with many of the previously confusing components clarified, and being more focused and better supported with the evidence presented, overall.

Thank you.

I have a few more comments:

Line 113: Regarding using DNA concentration as a proxy for biomass, I'm wondering if this was verified with more standard microbiological methods (e.g. OD measurement, CFU counts).

Because of the cloudy, particulate, "cricket media" that we used, we were not able to accurately measure O.D., and we chose not to use CFU counts since they were unlikely to capture all of the relevant species.

Line 213: Regarding "marginally higher" statement, the differences (Line 214) seem large to me.

We agree that the differences are not small in absolute terms, however, they are small relative to what we observe between replicates. We have changed the text accordingly: "However, while the cross-microcosm correlations of ASVs were moderately higher than those of any two random ASVs (mean = 0.23 and mode =0.32 vs. random pairs: mean = 0.003 and mode = 0.01, Figure 3d), species identity alone was not predictive of their dynamics; the community context was far more important. Indeed, when we compared ASV dynamics between filtered and unfiltered microcosms started from the same inoculum, we found much higher correlations (mean = 0.55 and mode = 0.89, Figure 3d), consistent with the notion that the dynamics in a given community context are deterministic."

Line 220-221: Please explain in more detail this new analysis. “Cosine similarity in ASV dynamics” is not very intuitive.

We now refer to the methods where we explain in detail how each analysis in Fig. 3c-e was performed: “For Fig. 3e, we measured the similarity in composition between communities by the Bray-Curtis distance on the untransformed relative abundance and the similarity between ASV dynamics by the cosine similarity between untransformed relative abundance time series. The cosine similarity metric uses the cosine of the angle between vectors and measures similarity irrespective of size. It was chosen in order to automatically remove time points where the ASV was not observed in one or both microcosms.”

Line 489-495: This seems unchanged from the first submission. Fig. 3e is different now.

Thank you for pointing out our error: this now refers to Supplementary Figure 3b, and we have made the appropriate edits.

Figure 3d: “Pick”. Is this a typo?

We have rewritten this part of the caption: “d) Probability density of correlation coefficients between the same ASVs in repetitions started from the same inocula, between the same ASVs in distinct microcosms, and between randomly chosen ASVs. Correlation coefficients are large when comparing replicate communities started from the same pitcher plant inocula. Between different microcosms, there is a moderate increase in the number of positive correlation coefficients relative to random pairs.”

Figure S2a: in the relative abundance graph, is this showing the filtered or unfiltered samples, or combined?

The relative abundance bar plot shows only the unfiltered samples. We have edited the figure legend to make this clear.

Figure S6: Please explain what/how this activity was measured/calculated. E.g., which enzymes were used?

We have added more information to this legend. It now reads: “Linear models indicate that strain endochitinase activity correlates with microcosm endochitinase activity, both across all microcosms, and within M03, M07 and M09. The natural logarithm of the enzyme activity was used, to better capture the broad spread of the activities. Endochitinase activity was measured using a Fluorimetric Chitinase Assay Kit as described in the methods.”

Reviewer #3 (Remarks to the Author):

Thank you for your work on the manuscript. I find it much improved and my comments

suitably addressed.
With one exception 394-401: Please explicitely state what the functional fingerprint of the Biolog EcoPlates actually means/measures (e.g. different C sources). Explain, what the dye response represents and how this differs from the MicroResp system.

Thank you for your comments. We have now added more information about the EcoPlate and MicroResp systems, to make clear how they differ and what they are measuring.
