# Supplementary material for: Context-dependent dynamics lead to the assembly of functionally distinct microbial communities
Source: Nat Commun. 2020 Mar 18;11:1440. doi: 10.1038/s41467-020-15169-0 (PMC7080782; doi:10.1038/s41467-020-15169-0)
Supplement: Supplementary file 2 — Peer Review File [file 41467_2020_15169_MOESM2_ESM.pdf]

Description of Additional Supplementary Files

File Name: Supplementary Data 1
Description: Information about cultured strains, including taxonomy, enzyme activity and BLAST hits.
